# *MYC*-driven aggressive B-cell lymphomas: biology, entity, differential diagnosis and clinical management

**DOI:** 10.18632/oncotarget.5774

**Published:** 2015-09-22

**Authors:** Qingqing Cai, L. Jeffrey Medeiros, Xiaolu Xu, Ken H. Young

**Affiliations:** ^1^ Department of Medical Oncology, Sun Yat-Sen University Cancer Center, State Key Laboratory of Oncology in South China, Collaborative Innovation Center of Cancer Medicine, Guangzhou, China; ^2^ Department of Hematopathology, The University of Texas MD Anderson Cancer Center, Houston, Texas, USA; ^3^ The University of Texas School of Medicine, Graduate School of Biomedical Sciences, Houston, Texas, USA

**Keywords:** MYC, Myc protein, translocation, microRNA, lymphoma

## Abstract

*MYC*, a potent oncogene located at chromosome locus 8q24.21, was identified initially by its involvement in Burkitt lymphoma with t(8;14)(q24;q32). *MYC* encodes a helix-loop-helix transcription factor that accentuates many cellular functions including proliferation, growth and apoptosis. *MYC* alterations also have been identified in other mature B-cell neoplasms and are associated with aggressive clinical behavior. There are several regulatory factors and dysregulated signaling that lead to *MYC* up-regulation in B-cell lymphomas. One typical example is the failure of physiological repressors such as Bcl6 or BLIMP1 to suppress *MYC* over-expression. In addition, *MYC* alterations are often developed concurrently with other genetic alterations that counteract the proapoptotic function of *MYC*. In this review, we discuss the physiologic function of *MYC* and the role that *MYC* likely plays in the pathogenesis of B-cell lymphomas. We also summarize the role *MYC* plays in the diagnosis, prognostication and various strategies to detect *MYC* rearrangement and expression.

## INTRODUCTION

*MYC* was recognized initially because of its involvement in t(8;14)(q24;q32) in Burkitt lymphoma, in which *MYC* at 8q24.21 is juxtaposed with *IGH* on the derivative chromosome 14 and causing *MYC* over-expression [1, 2]. *MYC* also can be overexpressed by juxtaposition with *IG* light chain loci, via t(2;8)(p11;q24) and t(8;22)(q24;q11), on the derivative chromosome 8 as well as with a number of non-IG gene loci. *MYC* is now known to be a potent oncogene and dysregulation of *MYC*, as a result of gene rearrangement or other mechanism, has been shown to be associated with extremely aggressive clinical behavior in lymphoma patients [1, 2]. However, *MYC* over-expression alone cannot cause lymphoma [3] and t(8;14)(q24;q32) also has been found at very low levels in the blood and bone marrow of apparently healthy individuals, suggesting that *MYC* alterations alone are insufficient to trigger lymphomagenesis. Burkitt lymphoma and other lymphomas that carry *MYC* translocations are highly proliferative tumors. In contrast, in normal germinal centers, the lymphoid cell compartment with the highest proliferative fraction where many *MYC* rearranged lymphomas originate, Myc expression is tightly controlled and it is difficult to identify Myc expression [1]. These findings also implicate other mechanisms that are essential for lymphomagenesis in *MYC* dysregulated lymphomas.

## BIOLOGIC AND PHYSIOLOGIC FUNCTIONS OF MYC

**Figure 1:**
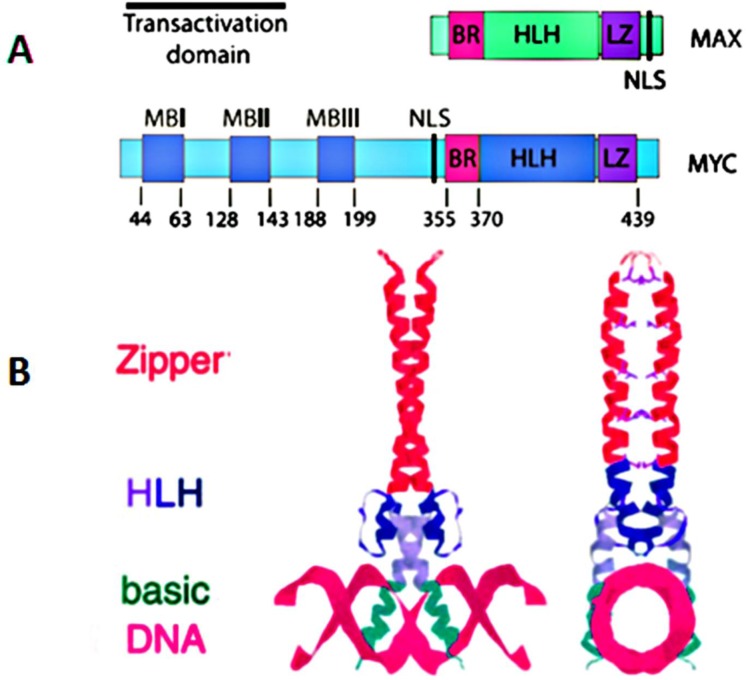
**A.**Functional domains of human c-*MYC* and its binding partner MAX; **B.** Structure of a Max homodimer bound to DNA.

**Figure 2:**
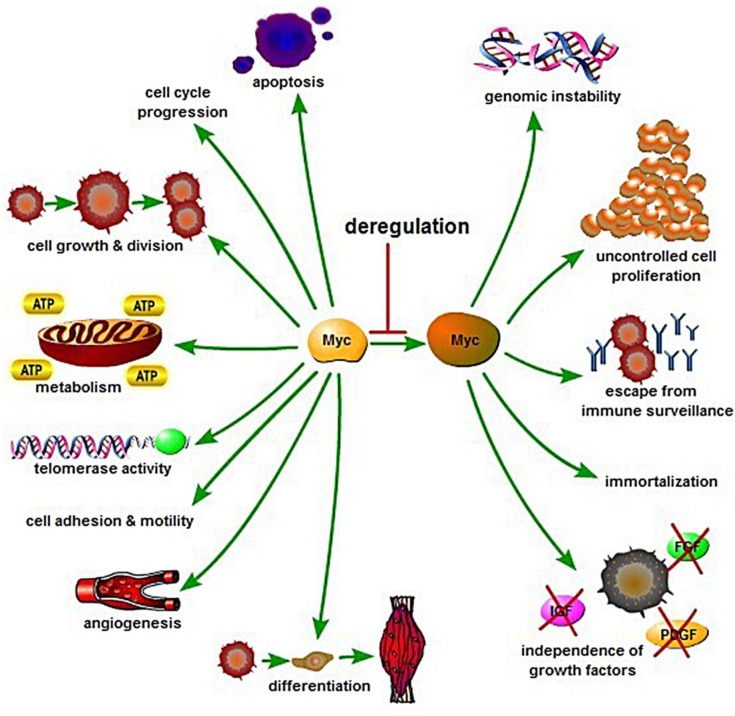
Biology and regulation of Myc in cellular processes control Myc is a key regulator of many biological activities including cell growth and division, cell-cycle progression, apoptosis, cell differentiation, cell metabolism, angiogenesis, cell adhesion and motility. Deregulation of Myc may result in apoptosis, genomic instability, uncontrolled cell proliferation, escape from immune surveillance, growth factor independence, and immortalization.

*MYC* is a basic helix-loop-helix transcription factor. Brodeur et al. found *MYC* in three forms, *MYC* (also known as C-*MYC*), *MYCL* and *MYCN* [4-6]. The *MYC*, *MYC*N (located at chromosome 2p24.3) and L-*MYC* (located at chromosome 1p34.2) genes encode transcription factors, i.e. proteins that bind to DNA and regulated transcription. *MYC* mRNA and Myc protein have very short half-life, approximately 10-25 minutes, respectively [7-10]. Myc polypeptides have N-terminal and a C-terminal regions (Figure 1). The C-terminal domain contains a basic HLH-Zip (helix-loop-helix-leucine zipper) domain. This terminal is a noncoding and allows Myc to dimerize with the related partner protein, Max (*MYC*-associated protein X). Dimerization of Myc and Max forms complexes that bind to the E-box, a starting site for transcription. The coding exons of *MYC* encode for the N-terminal region which has a transcriptional regulatory domain, a region that contains conserved *MYC* Boxes I and II, followed by *MYC* Box III and IV, and a nuclear targeting sequence. The N-terminal region will bind with co-activator complexes, making Myc act as the transcription or repression factor [1]. In this review we focus on *MYC* (*c-MYC*) which has been identified as a global transcription factor. It regulates more than 10% of genes in the human genome and plays essential roles in promoting cell growth, increasing cellular metabolism and mitochondrial biogenesis, and biosynthesis of nucleic acids, ribosomes, and protein, and has an important role in apoptosis (Figure 2) [1, 11].

*MYC* is considered as an oncogene because of its diverse biologic activity. The oncogenic potential of *MYC* in lymphomagenesis was first shown in transgenic mice in 1985 [12]. It has been shown that juxtaposing *MYC* with the immunoglobulin μ or κ enhancer in transgenic mice leads to the development of immature and mature B-cell neoplasms. Furthermore, several mechanisms for *MYC* deregulation in malignancy have been identified, including chromosome translocation, gene amplification, and insertional viral mutagenesis [2].

Chromosome translocation of *MYC* resulting in deregulation occurs most often in lymphoma types associated with aggressive clinical behavior, and in large part *MYC* deregulation accounts for the aggressive behavior [13]. Virtually all lymphomas with *MYC* deregulation are of B-cell lineage and include Burkitt lymphoma (BL), diffuse large B-cell lymphoma (DLBCL), B-cell lymphoma, unclassifiable with features intermediate between diffuse large B-cell lymphoma and BL (BCL-U), plasmablastic lymphoma, transformed follicular lymphoma, and rare *de novo* acute lymphoblastic lymphoma/leukemia (ALL).

## FUNCTIONAL REGULATION (TRANSCRIPTION, MICRO-RNAS AND APOPTOSIS)

### *MYC* as a transcription factor

*MYC* acts as a transcription factor by binding with Max [1, 4, 5], which depends on Enhancer Box (E-box) DNA sequence and recruitment of specific co-activator complexes [1, 3]. First, Myc-Max heterodimers start their activation of transcription by binding to the E-box [14]. After binding, transcriptional activation of *MYC* is mediated by binding to the histone acetyl-transferases, CBP/p300 and TIP60/GCN5, which requires the adaptor TRRAP, or the transcription factor P-TEFb/ubiquitin ligase SKP2, among others [15-17], resulting in transition from the G0/1 phase to the S phase. Myc also activates the expression of CCND2 (cyclin D2), cyclin-dependent kinases (CDKs) and down-regulates cell cycle inhibitors directly and indirectly. The cell phase transition ultimately induces cell proliferation and growth, DNA replication, protein biosynthesis, and regulation of metabolism and energy (Figure 2).

### *MYC* - micro-RNAs regulations

Apart from inducing cell proliferation and growth, the *MYC* transcriptional network regulates a large number of micro-RNAs (miRs) that function as oncogenes or tumor suppressor genes. MiRNAs are small (18-22 nt) noncoding RNAs that negatively regulate gene expression through the inhibition of translation and destabilization of messenger RNAs (mRNAs) [18]. *MYC* only up-regulates the oncogenic miR17-92-cluster [19]. The miR17-92-polycistron is commonly amplified at 13q31 in several subtypes of aggressive lymphomas [1, 3, 20]. Its oncogenic function is reflected by down-regulation of PTEN (phosphatase and tensin homolog deleted on chromosome ten), TP53 and E2F1, causing the activation of the PI3K/AKT pathway and inhibiting cellular apoptosis. *MYC* represses several miRs with tumor suppressor function by the recruitment of HDACs. These miRs are miR15a/16-1, miR26a, miR29, and miR34 that regulate important functions in the neoplastic development such as apoptosis (miR-15a/16-1 and miR-34 targeting *BCL2* and *TP53*, respectively), proliferation (miR-29a targeting CDK6) or cell differentiation (miR-26a targeting EZH2) [1, 21-23]. *MYC* is also negatively regulated by several miRs such as miR-34 and miR-494. MiR-494 is in turn repressed by EZH2 (enhancer of zeste homolog 2), making an auto-regulatory loop (*MYC*/miR-26a/EZH2/miR 494) that maintains persistent expression of *MYC* and EZH2 promoting the malignant phenotype (Figure 4) [1, 24].

**Figure 3:**
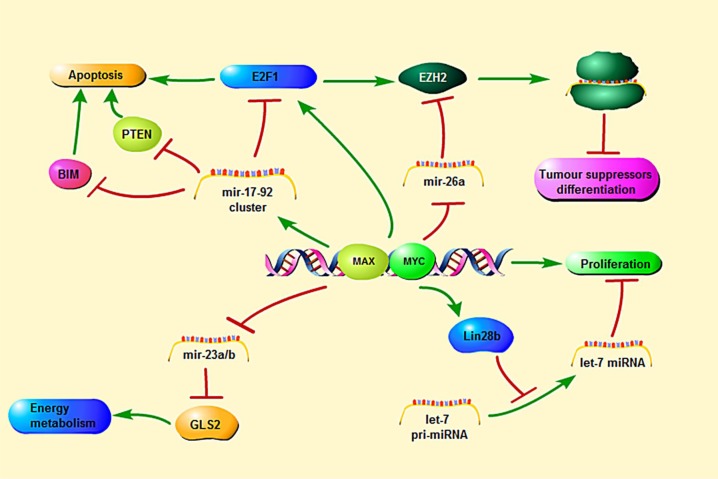
*MYC*-mediated regulation and signaling pathways relevant to transcription, microRNA and apoptosis

**Figure 4:**
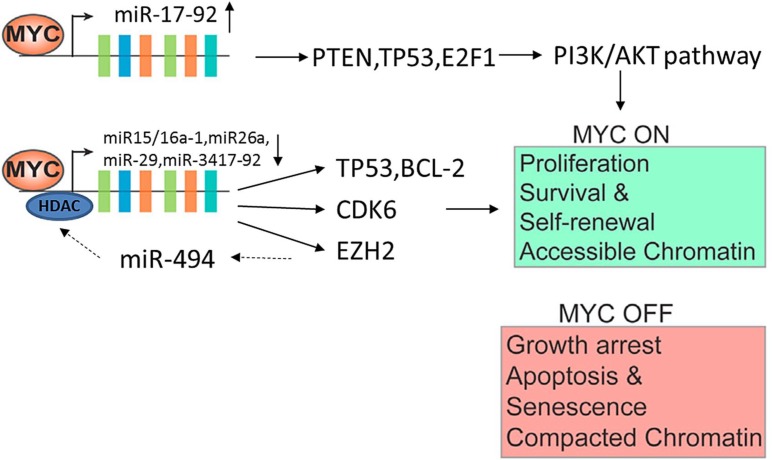
*MYC*-miRNA regulation in tumorgenesis The aggresivity in clinical behavior are induced by *MYC* upregulation together with several miRNA downregulation. The upregulation of miR 17-92 and downregulation of miR15a/16-1, miR26a, miR 29, and miR 34 are also involved. Deregulation of these *MYC*s may result in uncontrolled cell proliferation, escape from immune surveillance and immortalization.

Recent studies extend *MYC*'s broad activity to the entire non-coding transcriptome, including a larger and far less explored segment of the non-coding transcriptome, long non-coding RNAs (lncRNAs), which typically form functional secondary and higher order structures comprising protein-protein or protein-nucleic acid complexes [25]. LncRNAs are operationally defined as any non-protein-coding RNA > 200 nt in length, which corresponds to a convenient cut-off in biochemical fractionation and excludes all known classes of small RNAs [26]. Apart from encoding proteins, lncRNAs may have intrinsic functions as trans-acting regulatory RNAs, that in some contexts 3′UTRs can be separately expressed and convey genetic functions in trans, and that both may be further processed to produce subsidiary species [27]. The genome-wide effects of *MYC* on the non-coding transcriptome were discovered by RNAseq analysis. However, for selected genes, these data have been independently validated for specific lncRNAs by determining steady-state RNA levels by qRT-PCR and direct transcriptional effects by nuclear run-on experiments. This data is augmented by visualization with global epigenetic and *MYC* ChIP data [25].

*MYC* can also undergo transcriptional repression through several mechanisms. One mechanism is the interaction of these complexes with the transcription factor Miz-1 [1, 3]. This interaction prevents recruitment of the activating molecule p300 and facilitates the binding of the gene silencing DNA-methyltransferase, DNMT3a. Another mechanism of transcriptional repression occurs by transcription factors, such as Mad, titrating out Myc from the complexes allowing Max/Mad heterodimers to recruit histone deacetylases (HDAC) that cause repression of gene transcription. *MYC* may also repress gene expression by recruitment of HDAC to promoters containing E-boxes [1, 28].

The gene profile transcriptionally regulated by *MYC* varies in different cell types with relatively little overlap [29, 30]. Two recent studies showed that Myc, instead of activating a particular gene signature, can act as an amplifier of transcribed genes in a given cell by uploading to the promoters of active genes and enhancing their transcription. This function of *MYC* may be relevant to the increased aggressiveness of tumors associated with other oncogenic events carrying *MYC* alterations, and may offer for an opportunity to develop novel targeted therapies in the future [30, 31].

### *MYC* and apoptosis

In 2008, Hoffman & Liebermann demonstrated that elevated expression of *MYC* can induces apoptosis. Similar observations also were made for other oncogenic transcription factors such as E1A and E2F1 (Figure 3) [3, 32]. The biological meaning of this function is not fully understood, but has been interpreted as a cellular protective mechanism to counteract the effects of oncogenic activation and to avoid propagation of transformed cells.

The mechanisms of *MYC*-mediated apoptosis may involve several pathways. Overexpression of *MYC* increases DNA replication and possibly results in DNA damage that, in turn, triggers a *TP53*-mediated response leading to apoptosis [3, 33]. Myc expression also seems to downregulate, probably indirectly, anti-apoptotic proteins such as Bcl2 or Bcl-XL and upregulate pro-apoptotic elements such as BIM. This anti-tumorigenic effect of *MYC* may explain the need of other cooperative mechanisms for cell transformation and tumor progression.

There are some synthetic lethal interactions between Myc and some other molecular pathways. Identification of these *MYC* “synthetic lethal” pathways might facilitate therapeutic targeting of *MYC-*driven cancers. Recent studies indicate that BL cells are selected for B-cell receptor (BCR) expression. Given the essential role of the PI3K (phosphatidylinositol-4,5-bisphosphate 3-kinase) pathway in tonic BCR signaling, recent studies have addressed the potential cooperation of *MYC* and the BCR-dependent PI3K pathway [34]. Activation of PI3K pathway is also observed in human BL, especially in the sporadic subtype. Increased activity of the PI3K pathway in these tumors is a result of either gain of function mutations of TCF3 (transcription factor 3), or loss of function mutations of its negative regulator ID3 (inhibitor of DNA binding 3), that augment the BCR signaling [35]. The PIM family of oncogenic kinases is another group of proteins potentiating and consolidating *MYC*-dependent lymphomagenesis. PIMs directly phosphorylate and stabilize the Myc protein, but also prevent *MYC-*induced apoptosis by phosphorylation and inactivation of the proapoptotic protein BAD (*BCL2-*associated agonist of cell death) [36]. In addition, overexpression of *MYC* and Pims in mouse pre-B cells resulted in rapid proliferation but also an inhibition of differentiation, whereas genetic or pharmaceutical inhibition of Pim kinases decreased the proliferation of cells overexpressing Myc [35, 36].

The relevant oncogenic role of *MYC* has stimulated the search for therapeutic strategies that may counteract its damaging functions. Myc protein itself has been generally considered “undruggable” and therefore potential therapeutic approaches have been directed to reduce its expression, interfere with Max dimerization or DNA binding or acting on downstream target genes. However, most of these strategies have been difficult to apply *in vivo* in models [4, 37, 38]. The recent discovery that *MYC* transcription depends on the regulatory function of BRD4 offers new potential therapeutic opportunities [39, 40]. BRD4 is a member of the bromodomain and extraterminal (BET) subfamily of proteins that can bind to lysine acetylated histones and recruit elements required for transcription [1, 38, 39]. Two small molecules, JQ1 and iBET, displace BRD4 from acetylated chromatin resulting in downregulation of *MYC* and modulation of its transcriptional program, including the upregulation of *MYC* repressed miRNAs, with a marked anti-proliferative cell effect and tumor growth inhibition [1]. These results have been confirmed in plasma cell myeloma (PCM) and BL cell lines with translocated *IGH/MYC* and also in aggressive lymphomas with *MYC* overexpression not related to structural gene alterations, suggesting that this strategy may be useful in a broad spectrum of *MYC* driven tumors. Although BRD4 binds to a high number of enhancers and promoters, its inhibition is particularly sensitive in very large and active enhancers called super-enhancers that regulate oncogenes such as *MYC*. The addiction of PCM cells to *MYC* makes the cells particularly sensitive to the BRD4 binding disruption on its super enhancer [41].

## MYC REGULATION IN GERMINAL CENTER B-CELLS

*MYC* is initially expressed in B cells after interaction with antigens and T cells and is essential for germinal center (GC) formation [1, 11, 42]. Myc activates the cyclin-dependent kinase (CDK) complex, including cyclin D2 (CCND2). Through activation of this protein, Myc cause activation of cycle cell from the resting cell to become active cell.

After starting GC formation, Myc expression in GC is gradually suppressed by Bcl6 (B-cell lymphoma 6 protein). Bcl6, by binding with MIZ1 (ZBTB17), a known partner of *MYC*, represses transcription of *MYC* and inhibits CCND2 expression [1]. Switching gene expression from *MYC* to *BCL6* causes formation of the dark zone of GC. When Myc expression in dark zone GC is absent, TCF3 (E2A) acts as a transcription factor. TCF3 together with CCND3 acts in the dark zone of the GC to make these cells highly proliferative [43].

In the GC microenvironment, rapidly dividing B cells undergo somatic hypermutation and class-switch recombination of their immunoglobulin genes; both of these processes involve DNA strand breaks. These processes increase the probabil­ity of oncogenic events such as chromosomal rearrangements. In human GC-derived B cell lymphomas, the *MYC* gene is frequently involved in chromosomal translocations. Of note, it has been reported that cytidine deaminase (AID) is essential for the *MYC*/*IgH* chromosome translocations induced by IL6. AID is expressed in germinal center B cells, and most human B cell cancers represent germinal center or post- germinal center cells. *MYC/IgH* translocations in IL6 transgenic mice that are mutant for activation induced AID that initiates aberrant Ig class switch recombination [44].

TCF3 (E2A) not only acts as a transcription factor, but also induces its own negative inhibitor, ID3 [45]. ID3 inhibiting TCF3 causes the B-cell moving from the dark to the light zone. In this light zone, B-cells express IRF4 whereas Bcl6 is downregulated. Myc is re-expressed once the cells have been upregulated through NF-kB. This upregulation is again dependent on antigen and T-cell interactions.

The light zone *Myc*-positive cells have high-affinity BCRs and they can re-enter the dark zone, proliferate, and further acquire IG somatic mutations perpetuating the GC reaction. This is a highly interactive process. *Myc*-negative cells in the light zone exit the GC to become memory cells or early plasmablasts. Induction of BLIMP1 in plasmablasts causes plasma cell differentiation and represses Myc expression by binding to its promoter.

## GENOMIC AND EPIGENETIC ABERRATIONS

Gene amplification, translocations, point mutations, epigenetic reprogramming, enhanced translation and increased protein stability resulting in over-expression can induce *MYC* oncogenetic deregulation. *MYC* deregulation can be found in 80% of breast cancers, 70% of colon cancers, 90% of gynecological cancers, 50% of hepatocellular carcinomas, 30% of lung cancers and in many of hematological tumors.

Generally, *MYC* amplification has been described as the most frequent molecular alteration in most of solid tumors. Addition of single-nucleotide polymorphisms (SNPs) to the 8q24 chromosomal region has been associated with colorectal, bladder, breast, ovarian and prostate cancers.

The t(8;14)/*MYC-IGH* is the most frequent aberration involving the *MYC* gene in Burkitt lymphoma. Less common translocations involve the IG light chains, t(2;8)/*KAPPA-MYC* and t(8;22)/*MYC-LAMBDA* [1]. Activation of *MYC* gene is considered the main pathogenetic feature of Burkitt lymphoma, but other genetic mutations are also essential for lymphomagenesis. *MYC* rearrangement has been observed in 5-10% of diffuse large B-cell lymphomas and up to 50% of high-grade B-cell lymphomas other than Burkitt lymphoma. In these tumors, *MYC* translocations can also involve non-IG partners. Our recent study showed that *MYC* rearrangements are detected in 9% of diffuse large B-cell lymphomas. Patients with *MYC* rearranged diffuse large B-cell lymphomas more frequently present with bulky disease, fail to achieve complete remission with R-CHOP therapy and have poorer disease-specific survival, independent of the International Prognostic Index, if their tumors express Myc protein in more than half of the tumor cells [46]. Lu et al study showed that patients with *MYC* or *BCL2* copy number aberration (CNA) had significantly worse overall survival (OS) and progression-free survival (PFS) than negative cases (*P*<0.0001). Patients with both *MYC* and *BCL2* copy number aberration had similar outcomes to those with classic double-hit lymphoma (DHL) or protein double expression lymphoma (*MYC* and *BCL2*/*BCL6* coexpression) [47].

Myc protein overexpression as a result of point mutations in *MYC* N-terminal domain (residues 44-65) is also common. The most frequently mutated residue was Thr-58. Normally, the phosphorylation of Thr-58 can control Myc degradation and mutations. Mutation at Thr-58 causes an increase of Myc half-life in Burkitt lymphoma.

The mechanism by which Myc represses gene expression is less well understood. Schneider et al. reported that Myc acts to repress gene expression by binding with Miz-1. Wanzel et al. and Seoane et al. describe the role of Myc-Miz-1 interaction through TGFβ signaling pathway. In the absence of TGFβ, Myc represses CDKN2B by binding to Miz-1 and displacing Miz-1 cofactors. In the presence of TGFβ, Myc expression is suppressed. Then the Smad transcription factor translocates and binds with Miz-1, causing recruitment of Miz-1 cofactor, NPM1. The NPM1 recruitment causes stimulation of CDKN2B transcription, then induces cell cycle arrest. Myc also activates many ribosomal protein genes, one of which is Rpl23. This gene can bind to NPM1 in the nucleolus, and then cause inhibition of Miz-1 activity. Based on this description, NPM1 was described as a positive Myc coactivator [4, 48, 49].

Another critical mechanism for Myc-mediated gene repression is through its ability to activate microRNAs, resulting in E2F1 activity suppression, and TGFβ signaling pathway suppression [50]. Myc also represses many more microRNAs, resulting in increased gene expression at the protein level.

## DETECTION OF MYC DEREGULATION

Detection of *MYC* rearrangement has become an aid in the diagnosis of BL and PBL and a prognostic marker in other aggressive B-cell lymphomas. There are several techniques to detect *MYC* deregulation: conventional cytogenetics, fluorescence *in situ* hybridization (FISH), and immunohistochemistry (Table 1).

**Table 1:** Comparison of different detection techniques of *MYC*deregulation

Techniques	Method	Advantages	Disadvantages
Conventional cytogenetics [51, 52]	Assessing *MYC* translocations by determining the partner chromosome and gene, and can also determine whether the tumor has a simple or complex karyotype.	It is helpful to distinguishing some cases of BL from BCL-U. It is a very good and well-established method to survey the entire tumor genome.	It requires fresh viable tissue. A time-consuming and labor-intensive technique with a relatively long turnaround time (days to weeks). Requires skilled cytogenetic technologists and cytogeneticists to perform and interpret. It is only can be done at reference laboratories.
FISH [53]	Using fluorochrome-conjugated DNA complimentary to the *MYC* gene, to identify *MYC* gene rearrangement.	Can be performed on both fresh and formalin-fixed paraffin-embedded tissue. Shorter turn-around time (days).	Cannot identify the *MYC* translocation partner. Sometimes it can get false negatives result because the distribution of the breakpoints genomic region is large, approximately1000 kb. Require specialized personal to perform. It is only can be done at reference laboratories.
Immunohistochemistry [54]	Using rabbit monoclonal antibody (Epitomics, clone Y69) that targets the N-terminus of *MYC* to assess the differential expression of *MYC* in various aggressive B-cell lymphomas.	It can be performed quickly. It can be performed in most anatomic pathology laboratories. It can be assessed by a pathologist.	This method need more study to make the result become more valid.
CGH Array [55]	Comparing DNA extracted from the tumor to a commercially available pooled normal DNA control by using a high density 4×44K oligonucleotide array.	Can quantitatively detect single copy gains and losses and high level amplications in an experiment. Can detect these aberrations on a single clone.	It is unable to detect aberrations that do not result in copy number changes. It is limited in its ability to detect mosaicism.
Gene expression profile [56]	Transcriptome sequencing was carried out by RNA sequencing, and genes identified were validated using real-time PCR, which is used to amplify and simultaneously detect or quantify a targeted *MYC* DNA molecule.	RT-PCR allows quantification of the desired product at any point in the amplification process by measuring fluorescence.	Financial constraints limit expression profiling experiments to a small number of observations of the same gene under identical conditions, reducing the statistical power of the experiment.
Whole exome sequencing [57]	Sequencing all the protein-coding genes in a genome: Genomic DNA samples from patients were fragmented, ligated to Illumina multiplexing paired-end adapters, amplified by means of a PCR assay, and hybridized to biotin-labeled VCRome, a solution-based exome capture reagent that was designed in-house and is commercially available.	It is the most efficient way to identify the genetic variants in all of an individual's genes.	It is only able to identify those variants found in the coding region of genes which affect protein function. It's expensive relative to other technologies.
RPPA proteinic Array [58]	Sample cells are isolated from Myc patients and are lysed. The lysate is arrayed onto the microarray and probed with fluorescent antibodies against the target protein. Reference peptides are printed on the slides to allow for protein quantification of the sample lysates.	It is rapid, automated and highly sensitive, consuming small quantities of samples and reagents.	Proteins are more difficult to handle. A protein chip requires a lot more steps in its creation. It usually requires fresh reference peptides.

Karyotyping is a useful tool to assess for *MYC* translocations. This method can determine the partner chromosome involved in the translocation and also can determine whether the tumor has a simple or complex karyotype. A complex karyotype is gross evidence of other genetic abnormalities likely involved in lymphomagenesis or progression. This technique is helpful for distinguishing BL from BCL-U. However, when in daily practice, this technique has disadvantages. Conventional cytogenetics requires fresh, viable tissue and the results can take several days to up to 2-3 weeks to be available. This method also requires skilled cytogenetic technologists and cytogeneticists and is therefore usually performed in reference laboratories.

Fluorescence *in situ* hybridization (FISH) analysis is another useful technique to detect abnormalities of *MYC*. By using fluorochrome-conjugated DNA probes complementary to *MYC*, FISH can identify gene rearrangement using break-apart or dual fusion strategies. This technique can be performed on fresh viable tissue or formalin-fixed paraffin-embedded tissue sections and the turnaround time is shorter than conventional cytogenetics. However, FISH also has disadvantages. If a *MYC* break-apart probe is used, theoretically all gene rearrangements will be detected but this approach cannot provide information about the translocation partner. A second approach is a dual fusion strategy using probes specific for *IGH* and *MYC*. This approach detects t(8:14)/*MYC-IGH,* but cannot detect *IG light chain (IGL)* partners or non-IG *MYC* partners. (*Light chain* probes are not routinely available in most clinical laboratories.) In addition, dual fusion FISH sometimes can yield a false negative result if the distribution of the breakpoints is large around 1000 kb [53].

Another strategy for detecting *MYC* deregulation is immunohistochemistry using a novel rabbit monoclonal antibody (Epitomics, clone Y69) [54]. This reagent targets the N-terminus of *MYC* and can be used on paraffin-embedded tissue sections. Immunohistochemical assessment of Myc is advantageous in that it can be performed quickly and readily assessed in most anatomic pathology laboratories. Several recent studies have used this antibody to assess the differential expression of Myc in various aggressive B-cell lymphomas. Others have reported that nuclear or mixed nuclear and cytoplasmic staining for Myc correlates with *MYC* rearrangement. Ruzinova et al found that a primarily nuclear or mixed nuclear and cytoplasmic staining pattern for Myc in an aggressive B-cell lymphoma is highly predictive of a *MYC* translocation (positive-predictive value = 0.92, negative-predictive value = 0.95, *P* < 0.0001) and showed that the subcellular localization of Myc can be determined with good interobserver agreement among pathologists (k statistic = 0.90) [59]. Green et al analyzed the correlation between *MYC* rearrangement and Myc protein expression in 219 aggressive lymphomas in FISH and IHC study. In the study, 93% of cases with an *MYC* break had ≥80% Myc^+^ cells, in contrast to 3% of non-rearranged cases (*P* < 0.0001). Receiver operating characteristic curve analysis showed ≥70% Myc^+^ tumor cells to be the optimal cutoff sensitivity=100%, specificity=93%). Area under the receiver operating characteristic curve was 0.992, indicating that immunostaining for Myc protein is an excellent screening test to predict whether an *MYC* rearrangement is present [60]. Similarly, an international collaboration reported that only 11% of 167 patients with DLBCL had *MYC* translocations when using FISH, whereas IHC indicated that 29% of patients were positive for Myc protein expression [61]. Green et al show the value of simple immunohistochemical evaluation of *Myc* and *BCL2* expression for identifying patients with DLBCL with double-hit biology. Using this immunohistochemical approach, 29% of patients with DLBCL had DHL biology, which was strongly associated with poor prognosis after standard R-CHOP therapy [62-64]. However, immunohistochemistry is still not equivalent to the detection of *MYC* translocations [59, 61-64].

## MYC DYSREGULATION IN AGGRESSIVE LYMPHOMA, DIAGNOSIS AND CLASSIFICATION

*MYC* gene alterations were identified initially in lymphoid neoplasms by cytogenetic and molecular genetic studies that recognized 8q24 translocations and *MYC* gene rearrangements, amplifications, or mutations. These aggressive lymphomas also have additional oncogenic alterations that cooperate with *MYC* dysregulation by counteracting especially its proapoptotic function.

### Burkitt lymphoma (BL)

Burkitt lymphoma (BL) was first described by Denis Burkitt as a sarcoma involving the jaws of African children. Burkitt lymphoma is a mature B-cell lymphoma composed of very highly proliferating cells. Burkitt lymphoma has a germinal center B-cell (GCB) immunophenotype and is positive for the pan-B-cell antigens CD20, CD79a, and PAX5, and germinal center antigens CD10 and Bcl6.

**Figure 5:**
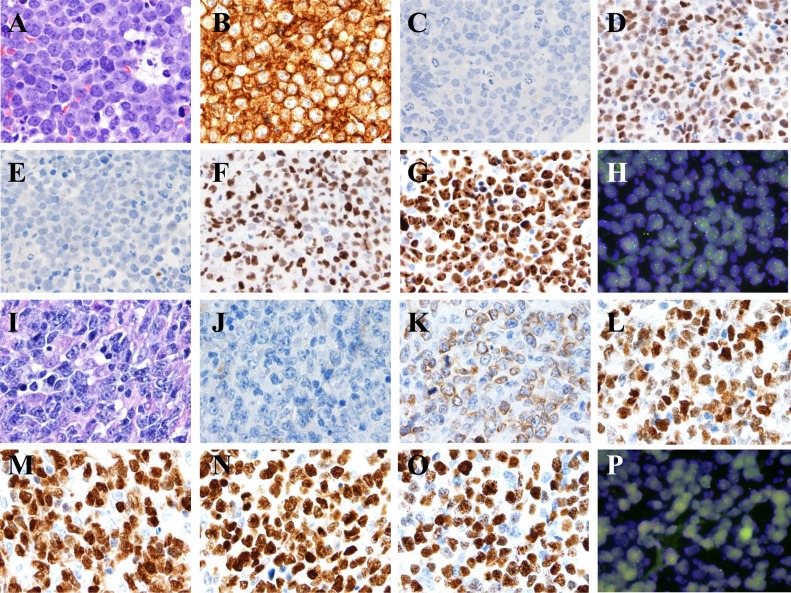

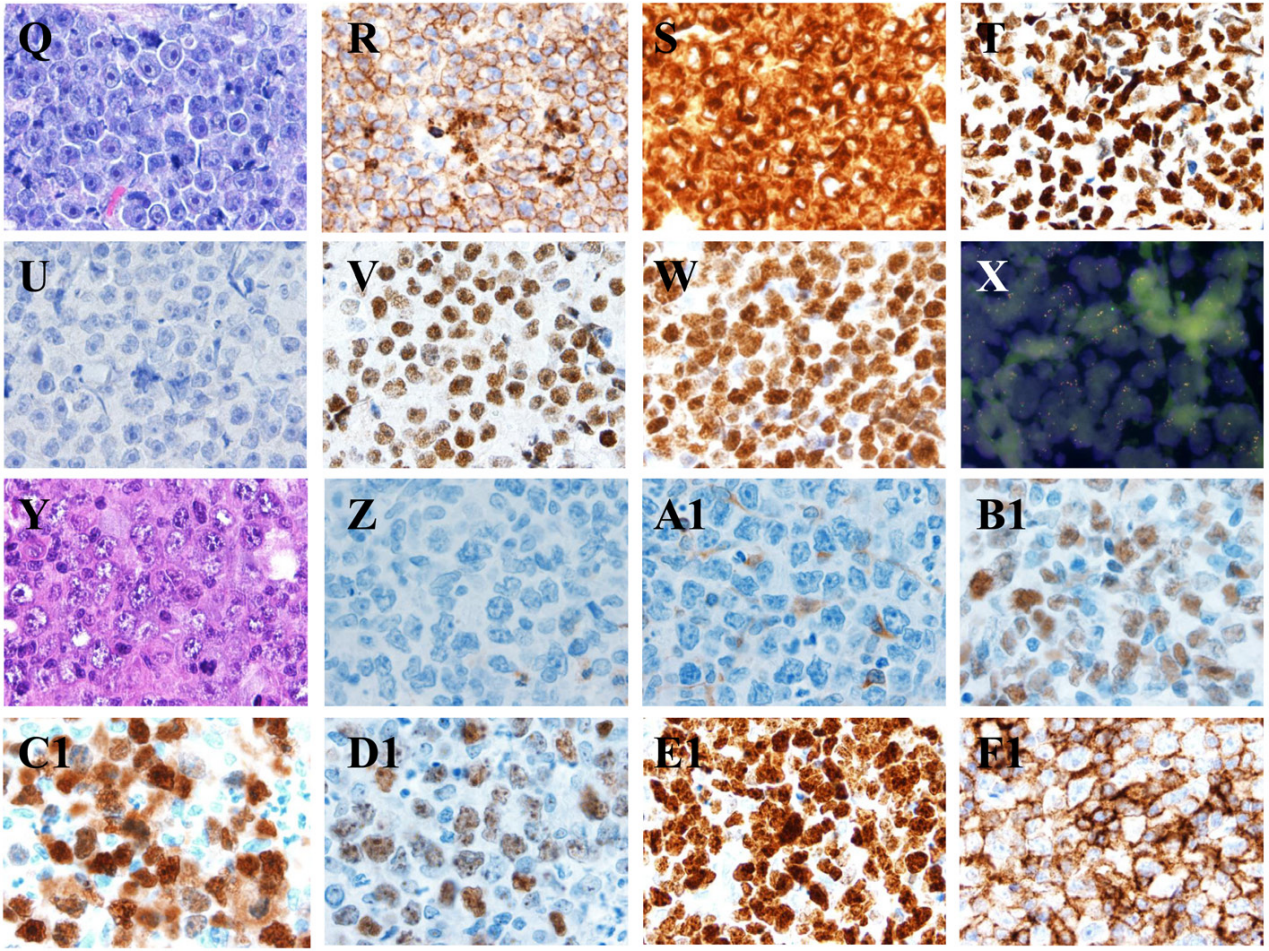
*MYC* translocations in aggressive B-cell lymphomas **A.**-**H.** Burkitt lymphoma. The lymphoma cells are monotonous in cell size with frequent apoptotic bodies and mitotic figures. Tingle-body macrophages are often seen with Starry-Sky pattern **A.** The tumor cells are positive for CD10 **B.**, BCL6 **D.**, FOXP-1 **F.**, high proliferation by Ki-67 **G.**, negative for BCL2 **C.** and MUM-1 **E.** All images, x 60 magnification. FISH showed t(8;14) as a sole cytogenetic abnormality **H.** x 100 magnification. **I.**-**P.** Diffuse large B-Cell Lymphoma, activated B-cell immunophenotype. The lymphoma cells are heterogeneous in cell size with occasional apoptotic bodies and mitotic figures. Reactive histiocytes and vascular proliferation are seen **I.** The tumor cells are positive for BCL2 **K.**, BCL6 **L.**, MUM-1 **M.**, FOXP-1 **N.**, high proliferation by Ki-67 **O.**, negative for CD10 **J.** All images, x 60 magnification. FISH showed non-IgH MYC gene translocation and disruption of MYC gene using break-part MYC probe **P.**, x 100 magnification. **Q.**-**X.** B-cell lymphoma unclassifiable with features intermediate between DLBCL and BL, **D.** Plasmablastic lymphoma. The lymphoma cells are relatively monotonous in cell size with frequent mitosis. Tingle-body macrophages are present but uncommon **Q.** The tumor cells are positive for CD10 **R.**, BCL2 **S.**, BCL6 **T.**, FOXP-1 **V.**, high proliferation by Ki-67 **W.**, negative for MUM-1 **U.**. All images, x 60 magnification. FISH showed a complex cytogenetic abnormality with t(8;14) and MYC gene amplification **X.**, x 100 magnification. (Y-F1) Plasmablastic lymphoma. The lymphoma cells are polymorphous in cell size with abundant cytoplasm. Reactive histiocytes and vascular proliferation are seen (Y). The tumor cells are positive for BCL6 (B1), MUM-1 (C1), FOXP-1 (D1), high proliferation by Ki-67 (E1), CD38 (F1), negative for CD10 (Z) and BCL2 (A1). FISH identified a complex cytogenetic abnormality with t(8;14). All images, x 60 magnification.

In the World Health Organization (WHO) classification, BL is subdivided into three variants: endemic BL (eBL), sporadic BL (sBL) and immunodeficiency-associated BL (iBL). The endemic variant can be found in young children at equatorial Africa, South America, and New Guinea. This variant is highly associated with Epstein-Barr virus (EBV) infection and usually occurs at extranodal sites, often involving facial bones and the abdomen. The sporadic variant is seen mostly in resource-rich nations throughout the world in adolescents and young adults. Sporadic BL is occasionally associated with EBV infection, about 20% of cases, and usually occurs within the abdomen, most frequently affecting the ileocecal region of the gastrointestinal tract. The immunodeficiency-associated variant is seen principally in patients infected with human immunodeficiency virus (HIV). About 30-40% of cases are associated with EBV infection. This variant can involve lymph nodes and extranodal sites; bone marrow involvement is more common in this variant [65]. The genetic hallmark of BL is *MYC* translocation (Figure 5) usually with the *IGH* locus but also with *IGL* loci (Table 2).

Beside from *MYC* translocations, *MYC* and *TP53* mutations also can be found in BL, in about 60% and 40% of the cases, respectively. Most of these mutations target functional domains that enhance the oncogenic potential of *MYC* by different mechanisms, including increased protein stability and transcriptional function or by impairing the induction of the proapoptotic element *BIM* [72].

**Figure 6:**
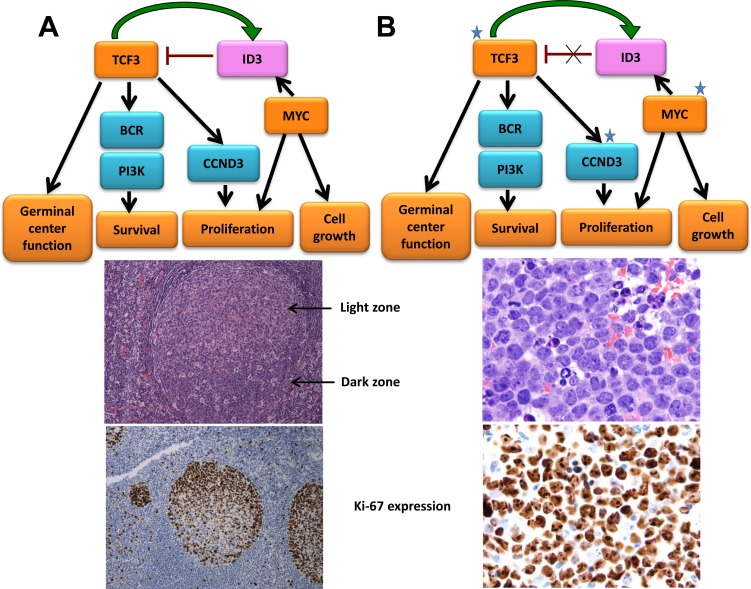
Somatic mutations in Burkitt lymphoma frequently target ID3, TCF3 and CCND3 **A.** TCF3 modulates germinal center genes and is preferentially expressed in the highly proliferative area of this structure, recognized histologically as the dark zone (DZ). TCF3 also regulates survival and proliferation of lymphoid cells through the BCR and PI3K signaling pathway and by modulating cell cycle regulators such as CCND3. TCF3 also induces its own inhibitor ID3, creating an autoregulatory loop that may attenuate this program and facilitate the transition of the germinal center cells to the light zone (LZ). *MYC* is not normally expressed in cells of the DZ and is upregulated in the LZ. Its induction of ID3 may contribute to the attenuation of the TCF3 pathway in the normal germinal center B-cells. Ki-67 shows active proliferation of DZ B-cells. **B.** Burkitt lymphoma (top) frequently harbors mutations in ID3, TCF3 and CCND3 that activate the TCF3 pathway. The t(8:14) translocation present in BL dysregulates *MYC*. Cooperation of these two pathways plays a crucial role in BL, in which virtually all cells are proliferating, as evidenced by the expression of the cell cycle–related antigen Ki-67 (bottom).

The gene expression profile of BL is similar to the cells in GC dark zone. This relationship seems contradictary since Myc is usually not expressed in the GC dark zone. *TCF3* and *ID3* mutations can be found in BL. The *ID3* mutations (38-68%) are more frequent than those of *TCF3* (11%), which have been found in about 70% of sporadic type and HIV-associated type BL but only 40% in endemic type BL. These mutations are important for the survival of BL cells, as *ID3* mutation causes constitutive TCF3 activation. Constitutive TCF3 activation strengthens BCR signaling through phosphoinositide-3-kinase (PI3K) pathway and CCND3 upregulation, causing cell survival and proliferation, respectively [43]. *CCND3* mutations can also be found in 38% of sporadic BL and occasional endemic tumors. Abnormalities in these three genes are important pathologic mechanisms involved in BL development (Figure 6).

**Table 2:** Aggressive B-cell lymphomas associated with recurrent MYC rearrangement

Lymphoma	Morphology	Karyotype	*MYC* Rearrangement	References
BL	Cohesive architecture Diffuse “starry-sky” proliferation of monomorphic medium-sized lymphocytes	Simple	Present in >90% of cases Translocations involve IG partner genes only 85% t(8;14):IGκ, IGλ 15% t(2;8),t(8;22) Not associated with *BCL2/BCL6* translocation	[66-68]
BCL-U	Often cohesive architecture “Intermediate” features of both DLBCL and BL	Complex	Present in 35%-50% of cases Translocations involve IG or non-IG partner genes, rarely involve IGH. Frequently associated with *BCL2/BCL6* translocation	[67, 68]
		
DLBCL	Variable architecture Diffuse proliferation of pleomorphic medium-to-large sized centroblastic, immunoblastic, or anaplastic lymphocytes	Complex	Present in 5%-14% of de novo cases Translocations involve IG or non-IG partner genes 50%-70% associated with *BCL2/BCL6* translocation	[35, 69, 70]
PBL	Diffuse proliferation of large immunoblastic and/or plasmacytic lymphocytes	Complex	Present in 50% of cases Translocations involve IG partner genes in most cases, usually IGH Not associated with*BCL2/BCL6* translocation	[71]

Note: BCL-U indicates B-cell lymphoma, unclassifiable with features intermediate between DLBCL and BL; BL, Burkitt lymphoma; DLBCL, diffuse large B-cell lymphoma; PBL, plasmablastic lymphoma.

However, molecular studies show that a small group of B cell lymphoma reminiscent of BL without *MYC* translocation carries the same GEP signature than BL. These cases are *MYC*-negative, high-grade B-cell lymphomas sharing a recurrent pattern of 11q aberration and similarities with but not being BL. Moreover, lymphomas with the typical 11q-gain/loss pattern seem to have more frequently nodal presentation than BL from patients younger than 40 years (82% vs 55%, *P*=0.074). The specific characteristics detected in this group of lymphomas raise the question of whether these lymphomas truly belong to the entity of BL and the presence of the typical 11q-gain/loss pattern could be a genetic hallmark that defines this group of cases [73].

Moreover, to define BL more precisely and to distinguish subgroups in other types of mature aggressive B-cell lymphomas, some studies used gene expression profiling to define a molecular BL (mBL) phenotype that would aid accurate diagnosis [74, 75]. Hummel et al devised a computational algorithm called “core-group extension.” Given a predefined core group of expression profiles that satisfied the WHO criteria for Burkitt's lymphoma (a consensus histologic classification of classic or atypical BL, CD20^+^, BCL6^+^, CD10^+^, BCL2^−^, CD5^−^, IG-myc^+^, and Ki-67 score ≥95%), the algorithm identified additional cases that have a similar pattern of gene expression and identified 58 genes that constituted the molecular Burkitt's lymphoma (mBL) signature. Each case was assigned an mBL-signature index score between 0 and 1, with a higher score reflecting a greater similarity of gene expression in the sample to that in the core group. Cases with an index score greater than 0.95 were classified as mBL. The distinctive mBL signature consisted of 58 genes, including several target genes of the NF-κB pathway (i.e., *BCL2A1, FLIP*, *CD44*, *NFKBIA*, *BCL3,* and *STAT3*) that are known to distinguish activated B-cell-like or germinal center B-cell-like lymphomas.

Using 95% similarity to a consensus mBL gene signature as the criterion, all classical BLs and almost all atypical BLs displayed an mBL phenotype, as did some (but not all) rare DLBCLs with ambiguous features [74]. A mBL represents a well-defined entity with very distinctive clinical and biological features which is well characterized by gene expression analysis, even if morphological features are inconsistent with BL.

### Diffuse large B-cell lymphoma

Diffuse large B-cell lymphoma (DLBCL) is the most common type of non-Hodgkin lymphoma in the world, accounting for 30% to 40% of all lymphoma cases. This disease usually occurs in adults and patients can present with nodal or extranodal sites of disease. Tumors consist of a diffuse proliferation of medium-large atypical lymphocytes that may resemble centroblasts, immunoblasts, or anaplastic cells. The neoplastic cells express pan B-cell antigens such as CD19, CD20, CD79a and PAX5 and variably express the anti-apoptosis protein Bcl2. Using immunohistochemistry and antibodies specific for CD10, Bcl6, MUM1, GCET1, and FOXP1 as a surrogate for gene expression profiling, DLBCL was divided into 2 subtypes, germinal center (GC), non-GCB; a subset of cases are unclassified [1, 65]. Dybkaer et al propose a refined classification system based on subset-specific B-cell-associated gene signatures (BAGS) in the normal B-cell hierarchy, hypothesizing that classification of the phenotypic cell of origin after gene signature assignment in malignant B-cell disorders should be assessed for clinical impact by end points, including diagnosis, prognosis, and prediction of therapeutic outcome [76].

Based on clinical and biological factors, several studies have examined the prognostic factors that may predict response to therapy. These factors include: the International Prognostic Index (IPI), concordant bone marrow involvement, Bcl2 expression, *BCL6* gene rearrangement and Bcl6 expression. Several studies have shown that patients with DLBCL with a GCB expression profile have a better prognosis and response to therapy than patients with DLBCL with a non-germinal center B-cell immunophenotype. 5-14% of DLBCL cases have been reported with *MYC* translocations (Figure 5). *MYC* rearrangement predicts for more aggressive disease, a poorer response to therapy, and is an independent predictor of overall survival in the rituximab era. About 2% of DLBCL cases have *MYC* amplification. Low copy number gains of *MYC* are more common in DLBCL (19-38%) and may be associated with higher levels of mRNA expression [77]. The presence of *MYC* translocation or amplification is most common in germinal center B-cell tumors that are positive for CD10 and Bcl6, and may be negative for Bcl2 (Table 1) [1, 67, 78-80].

*MYC* rearranged DLBCL may arise *de novo* or may represent at high-grade transformation of a low-grade lymphoma, in which case most commonly disease was follicular lymphoma (FL). In high grade transformation of FL, the *MYC* gene rearrangement is often accompanied by a t(14;18)(q32;q21) chromosome translocation/*BCL2* rearrangement. In the other studies, around 40% of patients, whose tumors carry a dual *MYC*/*BCL2* translocation, were reported to have a history of FL [81]. However, in other studies, 60-80% cases of DLBCL can be found with *MYC* rearrangements, accompanied by either *BCL2* or *BCL6* rearrangements, without history of low grade disease [1, 80, 82, 83].

*MYC* rearrangements in DLBCL can be partnered with *IGH* but, compared with Burkitt lymphoma, are more frequently partnered with the *IGL* or to non-*IG* genes such as *BCL6, BCL11A*, *PAX5* or *ICAROS49* [84]. *MYC* rearrangements in DLBCL are mainly found in patients > 60 years old with higher clinical stage, higher international prognostic index (IPI) values, and presentation of extranodal disease.

A *MYC* rearrangement in DLBCL is shown to be associated with inferior outcome in most studies. It is not clear whether the poor prognosis is attributable to the *MYC* rearrangement itself, or due to the fact that 58%-83% of *MYC* translocated DLBCL also had dual or even triple translocations that target *BCL2* and/or *BCL6.* High *MYC* amplifications copy number alterations also have been associated with shorter overall survival [79].

Myc protein expression is seen in most cases of DLBCL, but the number of positive cells varies from case to case. Myc protein is highly expressed (>70% of cells) in the nuclei of DLBCL with *MYC* rearrangements or amplification. However, only one third of DLBCL with substantial (>30-40% positive cells) Myc protein expression carry *MYC* gene alterations. This discordance suggests that mechanisms other than gene rearrangements are responsible for elevated protein expression in a considerable proportion of DLBCL cases. Myc overexpression has been associated with inferior prognosis in some studies [79]. As is the case with *MYC* translocations, Myc overexpression in DLBCL may not be predictive of an inferior prognosis on its own, since there is good evidence that it is the dual deregulation of both Myc and Bcl2 expression that is strongly correlated with shorter survival. Immunohistochemical expression scores using Myc, Bcl2, and possibly Bcl6, are able to identify patients with poor prognosis even within IPI subgroups [1, 61, 79].

### B-cell lymphoma, unclassifiable with features intermediate between diffuse large B-cell lymphoma and Burkitt lymphoma

B-cell lymphoma, unclassifiable, with features intermediate between DLBCL and BL (BCL-U) is a category in the 2008 WHO lymphoma classification. The reason WHO made it become one of B-cell lymphoma classification, is because it presents as a high-grade B-cell lymphoma but cannot be more precisely classified since it does not meet the criteria diagnostic for either BL or DLBCL. It seems likely that most of the tumors in this category are the high-grade end of the spectrum of DLBCL.

However, BCL-U tend to present in older adults with nodal or extranodal disease and advanced stage of disease. Histopathology of BCL-U is heterogeneous. Some cases closely resemble BL, whereas others are reminiscent of DLBCL. The neoplastic cells express the pan B-cell antigens CD19, CD20, CD79a, and PAX5. The BCL-U that resemble BL usually have an immunophenotype inconsistent with that diagnosis. Immunophenotypic features atypical for BL include the absence of Bcl6 expression, strong Bcl2 expression, and a lower (<95%) Ki-67 proliferation index. The BCL-U that resembles DLBCL may exhibit a BL immunophenotype. CD10 and Bcl6 are expressed. Bcl2 is absent, and the proliferation index is approximately 100%. This group of neoplasms is not associated with EBV infection. Interestingly, 30%-50% of BCL-U cases can carry *MYC* rearrangements (Figure 5) [1].

In contrast to BL, *MYC* rearrangements in BCL-U are frequently accompanied by translocations involving either *BCL2* and/or *BCL6*. The BCL-U with *MYC* accompanied either by *BCL2* or *BCL6* rearrangement or are referred as “double-hit“lymphoma (DHL). If all three genes are translocated, they are referred to as “triple-hit“ (TH) lymphoma. Patients with BCL-U that are also DH or TH lymphomas present with high clinical stage disease, high LDH, frequent extranodal manifestations and bone marrow and central nervous system infiltration. This type of disease had shorter average survival time, usually less than one year [83, 85-87]. *MYC* aberrations are also common in BCL-U. The presence of *MYC* aberrations identifies a patient subset that requires more aggressive therapy than R-CHOP. In contrast, *MYC* BCL-U patients responded variably to either R-CHOP or aggressive therapy, and the latter showed no survival advantage [88].

Recently, Momose et al sequenced relevant genes that are frequently mutated in BL (*ID3, TCF3*, *CCND3* and *MYC*) and DLBCL (*BCL2*, *EZH2*, *CREBBP*, *EP300*, *MEF2B* and *SGK1*) in 108 aggressive B-cell lymphomas including 31 BL, 24 BCL-U and 53 DLBCL cases, and found that the morphological, immunophenotypic and genetic gray zone between BL and DLBCL is also a gray zone of the mutational spectrum. Thus, it will be difficult to more precisely define molecular subgroups and relevant clinicopathologic entities within this spectrum of aggressive B-cell lymphomas [89].

### Double-hit and triple-hit lymphoma (DHL and THL)

The DH or TH genetic translocation is not restricted to DLBCL or BCL-U. It has also been observed in follicular lymphoma and B-cell lymphoblastic leukemia/lymphoma (TdT^+^). Most DH lymphomas had *MYC* and *BCL2* gene translocations, whereas a small subset has *MYC/BCL6* rearrangements [1, 78, 90].

In Mitelman's database of cytogenetic alterations in cancer, 62% of DH/TH cases had *MYC/BCL2* translocations. 16% of a triple-hit constellation involves *MYC*, *BCL2* and *BCL6*, and 8% of which involve *MYC/BCL6* rearrangement [91, 92]. More recently, *MYC/BCL6* rearrangement lymphomas have been reported to be more frequently CD10 negative with IRF4/MUM1 positive, and cytogenetically less complex than *MYC/BCL2* rearrangement lymphomas [93]. In general, in most studies, patients with DH/TH have been reported to run a dismal clinical course [94].

### Plasmablastic lymphoma

Plasmablastic lymphoma (PBL) is a rare aggressive B-cell neoplasm that commonly occurs in patients with immunodeficiency, including HIV-positive adults, posttransplant immunosuppressive therapy, iatrogenic immunosuppression for autoimmune disease, and immunosuppression associated with aging. A subset of patients is not apparently immunodeficient. Patients with these tumors commonly present with extranodal disease, with highest frequency in the head and neck region. The tumor can also affect bone, soft tissue, the gastrointestinal tract, skin, orbit, and the sinonasal cavities. Patients often show advanced stage and an intermediate to-high risk IPI score.

Characteristic cells of PBL are large, atypical with immunoblastic and/or plasmacytic features, and grow in a diffuse pattern. Mitotic figures are common, with highly proliferation index. The immunophenotype of malignant cells is plasmacytic, characterized by negative results for CD45, CD20, and PAX5 and positive results for CD38, CD138 and MUM1, with variably expression of CD79a and cytoplasmic immunoglobulins. Epstein-Barr virus (EBV) infection with latency I pattern of infection, in about 75% of cases [1, 81].

*MYC* translocations are also found in 41-49% of PBL (Figure 5). The translocation is commonly present as t(8;14) IGH/*MYC*. Rearrangements of *BCL6* or *BCL2* are rare and PBL does not seem to be part of the “double-hit” spectrum of disease [1, 81].

*MYC* activation also seems to play a role in the progression of plasma cell neoplasms particularly from monoclonal gammopathy of undetermined significance (MGUS) to plasma cell myeloma (PCM). This progression is associated with increased levels of Myc expression in the absence of structural alterations of the gene [95]. *MYC* rearrangements have been found in 0-15% of unselected PCM but in 45% of advanced tumors, particularly in those with extramedullary involvement, and in 65% of PCM cell lines, suggesting that *MYC* structural alterations are associated with progression of the tumors [71, 96, 97]. Contrary to PBL, *MYC* in PCM is frequently rearranged to non-*IGH* loci. The functional relevance of *MYC* in PCM has been highlighted by the addiction of these cells to *MYC* for survival.

Some patients with PBL and *MYC* rearrangements showed similar characteristic cells and immunophenotype with PCM [98]. But both of them can be differed by its association with an immunosuppressed state and presence of EBV infection, that can be found in PBL. Secondary PBL transformation from CLL (chronic lymphocytic leukemia) or FL also can be found *MYC* translocations [99, 100]. All these observations suggest that *MYC* aberrations do also play a role in the pathogenesis of aggressive lymphoid neoplasms with terminal B-cell differentiation. This finding is remarkable since most aggressive B-cell lymphomas with *MYC* rearrangements have a GC phenotype.

The terminal B-cell differentiation program is triggered by BLIMP1, a transcription factor highly expressed in PBL. BLIMP1 have several functions such as repressing gene characteristic for mature B-cell such as PAX5 genes, promoting gene that involves differentiation plasma cells such as XBP1 (X-Box binding protein 1), and repressing *MYC* and other controlling cell proliferation and cell growth genes. The presentation of *MYC* in this is maintained by activation of unfolded protein response (UPR), a protective antiapoptotic mechanism which is triggered in the endoplasmic reticulum that helps *MYC* to bypass the pro-apoptotic effect [1, 101].

### Lymphoblastic lymphoma

Lymphoblastic lymphoma is an aggressive non-Hodgkin lymphoma composed of lymphoblasts and an immature phenotype with expression of TdT and/or CD34 [65]. It is committed to the B-cell lineage (B-LBL) or T-cell lineage (T-LBL) that accounts for approximately 2% of all lymphomas [102]. Both T-LBL and B-LBL exist with different clinical phenotypes. T-LBL is much more common than B-LBL in adults, accounting for up to 90% of disease. It is typically seen in adolescents and young adults with a male predominance of nearly 3-fold [103, 104], mainly presents with a mediastinal mass and shortness of breath due to either compression of the superior vena cava or pericardial or pleural effusions. B-LBL has a more even age distribution with an older predominance [104, 105]. Compared with T-LBL, B-LBL is less likely to present with bone marrow (*P=*0.03) or mediastinal (*P=*0.04) involvement. Lymphoblastic lymphoma is a chemotherapy-sensitive disease with high initial response rates. However, relapse is common with conventional lymphoma regimens [106, 107]. One report suggests that B-LBL is more likely to achieve a complete remission (CR) than T-LBL (*P=*0.02), although it is limited by its retrospective nature and small numbers [105].

In T-LBL, neoplastic cells are usually TdT positive and variably express CD1a, CD2, CD3, CD4, CD5, CD7 and CD8. The only reliable lineage-specific is surface CD3. In B-LBL, tumour cells are virtually always positive for B cell markers CD19, CD79a and CD22. They are positive for CD10, CD 24, PAX5, and TdT in most cases, while the expression of CD20 and the lineage independent stem cell antigen CD34 is variable and CD45 may be absent. Surface immunoglobulin is usually absent. Most B-LBL have clonal rearrangements of the Ig heavy chain or less frequently of light chain genes. T-cell receptor γ or β chain gene rearrangements may be seen in a significant number of cases, but rearrangements are not helpful for lineage assignment [102].

In T-LBL, aberrant expression of *MYC* generally occurs downstream of activated NOTCH signaling, which is the major contributor to the pathogenesis of T-lymphoblastic malignancies [106-109]. Several retrospective studies showed that patients with PBC-LBL transformation from the preceding FL exhibited c-myc gene aberrations. Another study reports that 7 of 8 cases of transformed PBC-LBL are found with translocations involving 8q24.1 (c-myc gene locus) [110]. In addition, it is reported that LBL is characterised by concurrent *BCL2* and *MYC* rearrangements with IG gene(s), which may accelerate the onset of LBL tumorigenesis by suppressing Myc-induced apoptosis [111, 112].

### Transformed follicular lymphoma

Transformation of FL to DLBCL or t-FL occurs commonly. This disease has been associated with a poor prognosis. Firstly, these neoplastic cells express pan-B-cells antigens CD20, CD79a, and PAX5. Secondly, Davies et al found that 89% (31/35) of t-FL was of the GCB-DLBCL phenotype in which 80% (28/35) was CD10 positive, while 9% (3/35) were CD10 negative, BCL6 positive and MUM1 negative. Only 9% were classified as non-GCB phenotype with CD10 negative, BCL6 positive and MUM1 positive. Based on their report, no t-FL samples were of the ABC-like phenotype [113]. Thirdly, transformation of FL can occur in higher proliferation cancer types. These two different pathways are shown by expression of Myc. The transformation of *MYC* in t-FL can appear as the low frequency of translocations (*n* = 6/24 tFL with available FISH data, 25.0%), amplifications (*n* = 13/39, 33.3%) and point mutations reflecting the activity of aberrant somatic hypermutation which were the second commonest tFL-specific lesions gene [1, 114].

Lastly, transformation of FL can present *TP53* loss and mutation, *CDNK2A* loss and *c-REL* amplification. In the Davies et al report, 20% of t-FL patients had *wtTP53* in the antecedent FL. The recurrent alteration of genes involved in the control of cell cycle progression (*CDKN2A/B*, *MYC*) and DNA damage responses (alternative biallelic loss of *TP53* and *CDKN2A*) suggest that loss of genetic stability and deregulated proliferation are critical steps in tFL development. All of these mutations and amplifications caused the increasing proliferation rate of the tumor cells.

Beside these four characteristics of transformation follicular lymphoma, Lossos et al and Glas et al found increased expression of *LDHA* in this type of tumor. The other genes, such as *HK2*, *CENPF*, *CTPS*, *LDHA*, *P2RY5* and *DNASE1L3* also can be identified in both transformation follicular lymphoma and ‘indolent’ type FL [115, 116]. In a study by Gatenby et al, they found expression of the glycolytic genes (*HK2*, *PGM* and *LDHA*) reflects a shift in energy production from oxidative phosphorylation to aerobic glycolysis (the Warburg effect) which provides a complete growth advantage and is the basis of the increased 18F-fluorodeoxyglucose avidity observed by positron emission tomography post-transformation [113, 117].

### Mantle cell lymphoma

Mantle cell lymphoma (MCL) is a mature B-cell lymphoma characterized by t(11;14)(q13;q32)/*CCND1-IGH* and cyclin D1 overexpression [118, 119]. About 85% of cases are composed of a monotonous infiltrate of small lymphoid cells (centrocytes) without large cells (centroblasts). However, a subset of cases have cytologically aggressive cytologic features, resembling either lymphoblastic lymphoma (blastoid variant) or DLBCL (pleomorphic variant). A subset blastoid variant MCL cases carry *MYC* translocations, often involving *IGH*. These tumors can have BL-like morphologic features and patients have a very poor prognosis [120]. These tumors also can be considered as a form of DH lymphoma.

## TREATMENT IN AGGRESSIVE LYMPHOMA WITH MYC DYSREGULATION

**Figure 7:**
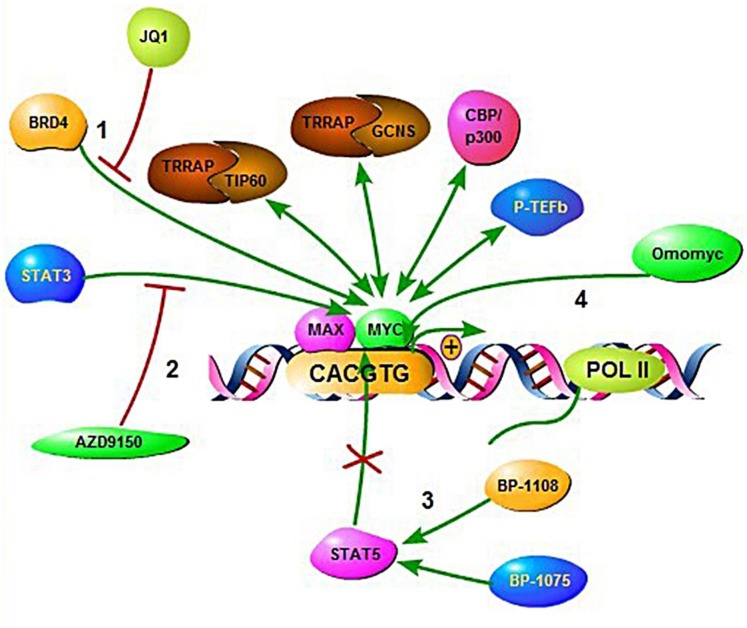
Illustration of therapeutic intervention approaches targeting *MYC* deregulated oncogenic pathways in aggressive B-cell lymphoma (1), In the murine study models of multiple myeloma manipulation of the BET bromodomain protein BRD4 using the compound JQ1. (2), Using a synthetic antisense nucleotide (ASO) to inhibit STAT3. (3), By binding at STAT5-SH2 domains using salicylic acid containing compounds. (4), Using Omo*MYC* to prevent *MYC* binding to promoter E-boxes.

*MYC* has been considered as an undruggable target because the protein structure does not respond to small-molecule inhibition. Wilson suggests two pathways to treat patients with aggressive lymphoma with *MYC* mutation. One approach is through manipulation of the BET bromodomain protein BRD4 and the other idea is through activating the transcription of tristetrapolin (Figure 7) [32, 38, 39, 121]. The BET Bromodomain has functions in the transcriptional machinery because it can recognize acetylated lysines in its histone tails. Because of that, bromodomain proteins serve as regulatory factors for Myc. In the murine study models of plasma cell myeloma, manipulation of the BET bromodomain protein BRD4 using the compound JQ1 can inhibit Myc gene expression (Table 3).

**Table 3:** New drugs in aggressive lymphoma with MYC dysregulation

Drug	Function
JQ1	inhibit *MYC* gene expression through manipulation BET bromodomain protein BRD4 [116]
BP-1108 and BP-1075	inhibit *MYC* gene expression through STAT5-SH2 binding domain [117]
AZD9150 (ISIS-STAT3Rx or ISIS 481464)	inhibit *MYC* gene expression through STAT3 [117]
Omomyc	inhibit *MYC* gene expression by prevent Myc binding to promoter E-boxes [118]

BET Bromodomain inhibitors also can induce tumor regression in mouse models of BL, AML, mixed lineage leukemia (MLL) and DLBCL [39, 40, 125]. Currently, the BET bromodomain inhibitor I-BET762 (GSK525762) is undergoing clinical phase I/II trial for relapsed/refractory hematologic malignancies patients. CPI-0610 and OTX015, other BET bromodomain inhibitors, are also undergoing phase I clinical trials in patients with hematologic malignancies [126]. Normal gene expression is tightly controlled by mRNA turnover, regulated by adenylate-uridylate-rich element (AU)-binding proteins (AUBP) that recognize AU-rich elements within transcripts. Tristetrapolin is an AUBP. Lymphomagenesis is promoted by Myc through suppressing the transcription of tristetrapolin. In lymphomas, this transcription is shown to be suppressed which causes no negative feedback in lymphomagenesis process. By restoring the tristetrapolin protein, it can prevent lymphomagenesis and abolishes the malignant transformation. Berg et al suggested another way to inhibit *MYC* over-expression by inhibiting the STAT5-SH2 binding domain [123]. STAT5, together with STAT1 and STAT3, regulate expression of genes that control the cell cycle (cyclin D1 and D2, and Myc), cell survival (Bcl-xL, Bcl-2, Mcl-1) and angiogenesis (HIF1α, VEGF). Berg et al found that chromone-derived nicotinylhydrazone, the most potent molecule, can disrupt the linkage between 5-carboxyfluorescein-GY (PO3H2) LVLDKW, derived from the erythropoietin (EPO) receptor, and the SH2 domain of STAT5b. This compound also inhibited IFNα stimulated STAT5 tyrosine phosphorylation in lymphoma cells. However, it still need high concentration of the compound (100-200 μM) to achieve the results. Gunning et al. continued the investigation and found more potent compounds to inhibit the STAT5-SH2 domain. Gunning and colleagues identified two salicylic acid based compounds, BP-1108 and BP-1075. These two compounds were potent *in vivo* inhibitors of STAT5 in MV-4-11 and K562 leukemia cell lines. BP-1108 also down-regulated STAT5 dependent genes, including *MYC*, *CCND1, CCND2*, and *MCL1*.

The other treatment was a synthetic antisense nucleotide (ASO) to inhibit STAT3, designated AZD9150 (ISIS-STAT3Rx or ISIS 481464). The ASO drugs function by binding with messenger RNA (mRNAs) and inhibiting production of disease-causing proteins. The drug already underwent phase I evaluation in six patients with advanced lymphoma (3 DLBCL, 2 Hodgkin lymphoma, 1 MCL) and nine patients with solid tumors. The result showed more than 50% reduction of tumor size in 2/3 DLBCL patients, and no responses in the patients with solid tumors. The drug is undergoing phase 2 clinical trials.

Savino et al suggested inhibition of Myc actionoperated through omomyc [32, 124]. Omomyc is a protein that is able to prevent Myc binding to promoter E-boxes and transactivation of target genes while retaining Miz-1 dependent binding to promoters and transrepression. This function was shown in an animal model of lung adenocarcinoma. The mice continuously received Omomyc and failed to develop lung adenocarcinoma without damaging normal tissues.

*MYC* also up-regulates genes that are involved in glycolysis and glutaminolysis for lymphoma growth. Several preclinical studies reported that inhibition of these enzymes can cause apoptosis and impair *MYC* function. For example, FX11 can elevate ROS production, reduce ATP levels, promote cell death and inhibit progression of *MYC*-dependent lymphoma xenografts through inhibition of lactate dehydrogenase A (LDHA) [127]. Inhibition of monocaroboxylate transporter by the specific inhibitor AZD3965 and inhibition of glutamine metabolism by the glutamate dehydrogenase 1 (GLUD1) inhibitor EGCG also caused cell apoptosis and repressed lymphoma growth [128-130]. Now AZD3965 and EGCG are undergoing phase I clinical trial in DLBCL patients and phase II clinical trial in multiple myeloma patients (Table 4).

**Table 4:** Current treatment strategy in aggressive lymphoma with MYC dysregulation

Compound name	Class	Target	References
Flavopiridol	CDK inhibitor	Cdk-9	Rahl et al. 2010 [125]
Purvalanol A	CDK inhibitor	Cdk-1	Goga et al. 2007 [126]
SU9516	CDK inhibitor	Cdk-2	Yu et al. 2002 [127]
PHA 767491 HCI	CDK inhibitor	Cdk-7 and Cdk-9	Montagnoli et al. 2008 [128]
SNS-032	CDK inhibitor	Cdk-2, Cdk-7, and Cdk-9	Chen et al. 2009 [129]
JQ1	BET bromodomain inhibitor	Bromodomain and Extra-Terminal motif (BET) proteins BRD2, BRD3, BRD4 and BRDT	Filippakopoulos et al. 2010 [130] Delmore et al. 2011 [39]
I-BET762 (GSK525762)	BET bromodomain inhibitor	Lipopolysaccharide-inducible gene	Chaidos et al. 2015 [120]
CPI-0610 and OTX015	BET bromodomain inhibitor	Bromodomain and Extra-Terminal motif (BET) proteins BRD2, BRD3, BRD4 and BRDT	Chaidos et al. 2015 [120]
SGI-1776	PIM kinase inhibitor	Pim-1	Zippo et al. 2007 and 2009 [131, 132]
EPZ004777	Dot1 L inhibitor	Dot1 L	Daigle et al. 2011 [133]
C464	p300/CBP ACTfrase inhibitor	p300	McMahon et al. 1998 [134]
SAHA	HDAC inhibitor	Histone deacetylases	Xu et al. 2005 [135]
Triptolide	TFIIH/XPB	XPB	Titov et al. 2011 [136]
Nutlin-3a	p53-MDM2 inhibitor	p53-MDM-2	Felsher et al. 2000 [137]
SB220025	MAPK inhibitor	p38	Campas et al. 2003 [138]
LY 294002	PI3K inhibitor	PI3K	Ikezoe et al. 2007 [139]
XAV-939	Wnt inhibitor	Tankyrase 1,2	Distler et al. 2013 [140]
LY-411575	γ-Secratase inhibitor	Notch 1	Moellering et al. 2009 [141]
VX-680	Aurora kinase inhibitor	Aurora kinases	Yang et al. 2010 [142]
NSC71948	BAG1 inhibitor	BAG-1	Zhang et al. 2011 [143]
ABT263	BCL2 inhibitor	BCL-2	Mason et al. 2008 [144]
VER-155008	HSP70 inhibitor	Hsp70/Hsc70	Goloudina et al. 2012 [145]
KW-2478	HSP90 inhibitor	Hsp90	Nakashima et al. 2010 [146]
SB 218078	Chk1 inhibitor	Chk-1	Murga et al. 2011 [147]
Leflunomide	DHODH inhibitor	DHODH	Liu et al. 2007 [148]
BP-1108, BP-1075	STAT5 inhibitor	STAT5-SH2 domain	Page et al [149]
AZD9150	STAT3 inhibitor	STAT3	Hong et al. 2013 [150] Furqan et al. 2013 [117]
AZD3965	Monocaroboxylate transporter inhibitor	Monocaroboxylate transporter	Li et al. 2013 [124]
EGCG	GLUD1 inhibitor	Glutamate dehydrogenase	Qing et al. 2012 [123]

In light of poor outcomes with *MYC*-related lymphomas, both intensified chemoimmunotherapy regimens and consolidative stem cell transplantation (SCT) have been evaluated for *MYC* management. Cuccuini et al determined the prognostic significance of *MYC* rearrangement in patients with relapsed/refractory DLBCL prospectively treated by R-ICE or R-DHAP followed by high-dose therapy and autologous stem cell transplantation. The 4-year PFS and OS were significantly lower in the *MYC* ^+^ DLBCL patients than those in the *MYC* ^−^ DLBCL patients. Type of treatment, R-DHAP or R-ICE had no impact on survivals. Among 161 available tissue specimens, 28 (17%) had *MYC* rearrangements; but, notably, they did not have a higher frequency of early relapse from induction R-CHOP. The lack of a clear benefit from intensification of therapy (with or without SCT) suggests that incorporating novel and targeted agents should be pursued, and several candidate agents are available [156].

Currently, combination chemotherapy with R-EPOCH is the only treatment regimen that has the potential to induce complete response in a proportion of DHL patients, although with no overall survival advantage [157]. Howlett et al compared survival outcomes in DHL patients and found out first-line treatment with R-EPOCH significantly reduced the risk of a progression compared with R-CHOP (relative risk reduction of 34%; *P =* 0.032); however, overall survival (*n* = 374) was not significantly different across treatment approaches. A subset of patients might benefit from intensive induction with/without transplant. Results of these pooled meta-analytic estimates suggest that higher-dose regimens may overcome the factors associated with poor risk in the in DLBCL and FL patients harbouring rearrangements of the *MYC* and *BCL2* genes [158].

In a retrospective analysis of 52 patients with *MYC*/*BCL2* double-hit lymphoma, Li et al reported a median overall survival of 18.6 months. The majority of patients in this study received high-intensity chemotherapy regimens (primarily R-hyper-CVAD), while the remaining received a moderate-intensity regimen (R-CHOP). 21% of patients received consolidation with either allogeneic stem cell transplantation or high-dose chemotherapy with autologous stem cell rescue. While elevated LDH, >1 extranodal site and IPI>2 were associated with shorter survival in univariate analysis, neither intensity of treatment regimen nor stem cell transplantation impacted outcomes [87]. Further investigation into the role of transplant and novel therapy combinations is necessary. The clinical exploration of front-line targeted agents represents a reasonable approach to developing novel treatment options for this patient population [159-162].

### Conclusions and future directions

*MYC* is a transcription factor that influences cell activity including cell growth, cell cycle progression, survival and biosynthesis, or induces apoptosis. *MYC* translocation was first found in Burkitt lymphoma, that carries t(8;14)(q24;q32). *MYC* translocation is not the only feature in Burkitt lymphoma, as mutation in TCF3/ID3 pathway has also been found in this disease. *MYC* translocation can also be found in DLBCL, singly or together with translocations involving *BCL2* or *BCL6*. *MYC* translocations are also involved in other rare aggressive lymphoma types as well as in transformation of low-grade lymphomas. Others have identified several mechanisms to inhibit expression of *MYC*. Firstly, inhibiting Myc expression by manipulating the BET bromodomain protein BRD4. Secondly, inhibiting Myc expression through STAT5-SH2 binding domain or STAT3; and lastly inhibiting Myc expression by preventing Myc binding to promoter E-boxes. However, the clinical utility of strategies targeting *MYC* for patients with lymphoid malignancy remains to be better defined. A complete understanding of the mechanisms by which *MYC* achieves these oncogenic effects remains a daunting challenge. Future researches could include combinations with existing therapies, careful planning of study protocols and the development of unique biomarkers targeted *MYC* signaling pathway. It is also a task that promises to provide critical new insights into the molecular biology of *MYC*-related malignancy.

## References

[1] Ott G, Rosenwald A, Campo E (2013). Understanding MYC-driven aggressive B-cell lymphomas: pathogenesis and classification. Blood.

[2] Meyer N, Penn LZ (2008). Reflecting on 25 years with MYC. Nat Rev Cancer.

[3] Klapproth K, Wirth T (2010). Advances in the understanding of MYC-induced lymphomagenesis. Br J Haematol.

[4] Dang CV (2012). MYC on the path to cancer. Cell.

[5] Lüscher B, Vervoorts J (2012). Regulation of gene transcription by the oncoprotein MYC. Gene.

[6] Aquino G, Marra L, Cantile M, De Chiara A, Liguori G, Curcio MP, Sabatino R, Pannone G, Pinto A, Botti G (2013). MYC chromosomal aberration in differential diagnosis between Burkitt and other aggressive lymphomas. Infect Agent Cancer.

[7] Kelly K, Cochran BH, Stiles CD, Leder P (1983). Cell-specific regulation of the c-myc gene by lymphocyte mitogens and platelet-derived growth factor. Cell.

[8] Dani C, Blanchard J, Piechaczyk M, El Sabouty S, Marty L, Jeanteur P (1984). Extreme instability of myc mRNA in normal and transformed human cells. Proc Natl Acad Sci USA.

[9] Hann SR, Eisenman RN (1984). Proteins encoded by the human c-myc oncogene: differential expression in neoplastic cells. Mol Cell Biol.

[10] Hann SR (2006). Role of post-translational modifications in regulating c-Myc proteolysis, transcriptional activity and biological function. Semin Cancer Bio.

[11] Calado DP, Sasaki Y, Godinho SA, Pellerin A, Köchert K, Sleckman BP, de Alborán IM, Janz M, Rodig S, Rajewsky K (2012). The cell-cycle regulator c-Myc is essential for the formation and maintenance of germinal centers. Nat Immunol.

[12] Adams J, Harris A, Pinkert C, Corcoran L, Alexander W, Cory S, Palmiter R, Brinster R (1984). The c-myc oncogene driven by immunoglobulin enhancers induces lymphoid malignancy in transgenic mice. Nature.

[13] Johnson NA, Savage KJ, Ludkovski O, Ben-Neriah S, Woods R, Steidl C, Dyer MJ, Siebert R, Kuruvilla J, Klasa R (2009). Lymphomas with concurrent BCL2 and MYC translocations: the critical factors associated with survival. Blood.

[14] Uribesalgo I, Buschbeck M, Gutiérrez A, Teichmann S, Demajo S, Kuebler B, Nomdedéu JF, Martín-Caballero J, Roma G, Benitah SA (2011). E-box-independent regulation of transcription and differentiation by MYC. Nat Cell Biol.

[15] Kanazawa S, Soucek L, Evan G, Okamoto T, Peterlin BM (2003). c-Myc recruits P-TEFb for transcription, cellular proliferation and apoptosis. Oncogene.

[16] Kim SY, Herbst A, Tworkowski KA, Salghetti SE, Tansey WP (2003). Skp2 regulates Myc protein stability and activity. Mol Cell.

[17] Gargano B, Amente S, Majello B, Lania L (2007). P-TEFb is a crucial co-factor for Myc transactivation. Cell Cycle.

[18] Psathas JN, Doonan PJ, Raman P, Freedman BD, Minn AJ, Thomas-Tikhonenko A (2013). The Myc-miR-17-92 axis amplifies B-cell receptor signaling via inhibition of ITIM proteins: a novel lymphomagenic feed-forward loop. Blood.

[19] Chang T-C, Yu D, Lee Y-S, Wentzel EA, Arking DE, West KM, Dang CV, Thomas-Tikhonenko A, Mendell JT (2008). Widespread microRNA repression by Myc contributes to tumorigenesis. Nat Genet.

[20] Navarro A, Beà S, Fernández V, Prieto M, Salaverria I, Jares P, Hartmann E, Mozos A, López-Guillermo A, Villamor N (2009). MicroRNA expression, chromosomal alterations, and immunoglobulin variable heavy chain hypermutations in Mantle cell lymphomas. Cancer Res.

[21] Zhang X, Zhao X, Fiskus W, Lin J, Lwin T, Rao R, Zhang Y, Chan JC, Fu K, Marquez VE (2012). Coordinated silencing of MYC-mediated miR-29 by HDAC3 and EZH2 as a therapeutic target of histone modification in aggressive B-Cell lymphomas. Cancer Cell.

[22] Sotillo E, Laver T, Mellert H, Schelter J, Cleary M, McMahon S, Thomas-Tikhonenko A (2011). Myc overexpression brings out unexpected antiapoptotic effects of miR-34a. Oncogene.

[23] Bueno MJ, Malumbres M (2011). MicroRNAs and the cell cycle. Biochim Biophys Acta.

[24] Li Y, Choi PS, Casey SC, Dill DL, Felsher DW (2014). MYC through miR-17-92 suppresses specific target genes to maintain survival, autonomous proliferation, and a neoplastic state. Cancer cell.

[25] Weinberg MS, Hart JR, Vogt PK (2015). A brave new MYC-amplified world. Aging (Albany NY).

[26] Mercer TR, Dinger ME, Mattick JS (2009). Long non-coding RNAs: insights into functions. Nat Rev Genet.

[27] Morris KV, Mattick JS (2014). The rise of regulatory RNA. Nat Rev Genet.

[28] Zhang X, Chen X, Lin J, Lwin T, Wright G, Moscinski L, Dalton W, Seto E, Wright K, Sotomayor E (2012). Myc represses miR-15a/miR-16-1 expression through recruitment of HDAC3 in mantle cell and other non-Hodgkin B-cell lymphomas. Oncogene.

[29] Littlewood TD, Kreuzaler P, Evan GI (2012). All things to all people. Cell.

[30] Nie Z, Hu G, Wei G, Cui K, Yamane A, Resch W, Wang R, Green DR, Tessarollo L, Casellas R (2012). c-Myc is a universal amplifier of expressed genes in lymphocytes and embryonic stem cells. Cell.

[31] Lin CY, Lovén J, Rahl PB, Paranal RM, Burge CB, Bradner JE, Lee TI, Young RA (2012). Transcriptional amplification in tumor cells with elevated c-Myc. Cell.

[32] Miller DM, Thomas SD, Islam A, Muench D, Sedoris K (2012). c-Myc and cancer metabolism. Clin Cancer Res.

[33] Vousden KH, Prives C (2005). P53 and prognosis: new insights and further complexity. Cell.

[34] Srinivasan L, Sasaki Y, Calado DP, Zhang B, Paik JH, DePinho RA, Kutok JL, Kearney JF, Otipoby KL, Rajewsky K (2009). PI3 kinase signals BCR-dependent mature B cell survival. Cell.

[35] Sewastianik T, Prochorec-Sobieszek M, Chapuy B, Juszczynski P (2014). MYC deregulation in lymphoid tumors: molecular mechanisms, clinical consequences and therapeutic implications. Biochim Biophys Acta.

[36] Fox CJ, Hammerman PS, Cinalli RM, Master SR, Chodosh LA, Thompson CB (2003). The serine/threonine kinase Pim-2 is a transcriptionally regulated apoptotic inhibitor. Genes Dev.

[37] Berg T (2011). Small-molecule modulators of c-Myc/Max and Max/Max interactions. Curr Top Microbiol Immunol.

[38] Albihn A, Johnsen JI, Henriksson MA (2010). MYC in oncogenesis and as a target for cancer therapies. Adv Cancer Res.

[39] Delmore JE, Issa GC, Lemieux ME, Rahl PB, Shi J, Jacobs HM, Kastritis E, Gilpatrick T, Paranal RM, Qi J (2011). BET bromodomain inhibition as a therapeutic strategy to target c-Myc. Cell.

[40] Mertz JA, Conery AR, Bryant BM, Sandy P, Balasubramanian S, Mele DA, Bergeron L, Sims RJ (2011). Targeting MYC dependence in cancer by inhibiting BET bromodomains. Proc Natl Acad Sci USA.

[41] Lovén J, Hoke HA, Lin CY, Lau A, Orlando DA, Vakoc CR, Bradner JE, Lee TI, Young RA (2013). Selective inhibition of tumor oncogenes by disruption of super-enhancers. Cell.

[42] Dominguez-Sola D, Victora GD, Ying CY, Phan RT, Saito M, Nussenzweig MC, Dalla-Favera R (2012). The proto-oncogene MYC is required for selection in the germinal center and cyclic reentry. Nat Immunol.

[43] Schmitz R, Young RM, Ceribelli M, Jhavar S, Xiao W, Zhang M, Wright G, Shaffer AL, Hodson DJ, Buras E (2012). Burkitt lymphoma pathogenesis and therapeutic targets from structural and functional genomics. Nature.

[44] Ramiro AR, Jankovic M, Eisenreich T, Difilippantonio S, Chen-Kiang S, Muramatsu M, Honjo T, Nussenzweig A, Nussenzweig MC (2004). AID is required for c-myc/IgH chromosome translocations *in vivo*. Cell.

[45] Seitz V, Butzhammer P, Hirsch B, Hecht J, Gütgemann I, Ehlers A, Lenze D, Oker E, Sommerfeld A, von der Wall E (2011). Deep sequencing of MYC DNA-binding sites in Burkitt lymphoma. PLoS One.

[46] Tzankov A, Xu-Monette ZY, Gerhard M, Visco C, Dirnhofer S, Gisin N, Dybkaer K, Orazi A, Bhagat G, Richards KL (2014). Rearrangements of MYC gene facilitate risk stratification in diffuse large B-cell lymphoma patients treated with rituximab-CHOP. Mod Pathol.

[47] Lu TX, Fan L, Wang L, Wu JZ, Miao KR, Liang JH, Gong QX, Wang Z, Young KH, Xu W, Zhang ZH, Li JY (2015). MYC or BCL2 copy number aberration is a strong predictor of outcome in patients with diffuse large B-cell lymphoma. Oncotarget.

[48] Cascón A, Robledo M (2012). MAX and MYC: a heritable breakup. Cancer Res.

[49] Soucek L, Evan GI (2010). The ups and downs of Myc biology. Curr Opin Genet Dev.

[50] Mestdagh P, Bostrom AK, Impens F, Fredlund E, Van Peer G, De Antonellis P, von Stedingk K, Ghesquiere B, Schulte S, Dews M, Thomas-Tikhonenko A, Schulte JH, Zollo M, Schramm A, Gevaert K, Axelson H (2010). The miR-17-92 microRNA cluster regulates multiple components of the TGF-beta pathway in neuroblastoma. Mol Cell.

[51] Wang YL, Bagg A, Pear W, Nowell PC, Hess JL (2001). Chronic myelogenous leukemia: laboratory diagnosis and monitoring. Genes Chromosomes Cancer.

[52] Carey CD, Gusenleitner D, Chapuy B, Kovach AE, Kluk MJ, Sun HH, Crossland RE, Bacon CM, Rand V, Dal Cin P, Le LP, Neuberg D, Sohani AR, Shipp MA, Monti S, Rodig SJ (2015). Molecular classification of MYC-driven B-cell lymphomas by targeted gene expression profiling of fixed biopsy specimens. J Mol Diagn.

[53] Haralambieva E, Schuuring E, Rosati S, van Noesel C, Jansen P, Appel I, Guikema J, Wabinga H, Bleggi Torres L Fernando, Lam K (2004). Interphase fluorescence *in situ* hybridization for detection of 8q24/MYC breakpoints on routine histologic sections: validation in Burkitt lymphomas from three geographic regions. Genes Chromosomes Cancer.

[54] Gurel B, Iwata T, Koh CM, Jenkins RB, Lan F, Van Dang C, Hicks JL, Morgan J, Cornish TC, Sutcliffe S (2008). Nuclear MYC protein overexpression is an early alteration in human prostate carcinogenesis. Mod Pathol.

[55] Jardim DL, Conley A, Subbiah V (2013). Comprehensive characterization of malignant phyllodes tumor by whole genomic and proteomic analysis: biological implications for targeted therapy opportunities. Orphanet J Rare Dis.

[56] Louro ID, Bailey EC, Li X, South LS, McKie-Bell PR, Yoder BK, Huang CC, Johnson MR, Hill AE, Johnson RL, Ruppert JM (2002). Comparative gene expression profile analysis of GLI and c-MYC in an epithelial model of malignant transformation. Cancer Res.

[57] Ng SB, Turner EH, Robertson PD, Flygare SD, Bigham AW, Lee C, Shaffer T, Wong M, Bhattacharjee A, Eichler EE, Bamshad M, Nickerson DA, Shendure J (2009). Targeted Capture and Massively Parallel Sequencing of Twelve Human Exomes. Nature.

[58] Petricoin EF, Zoon KC, Kohn EC, Barrett JC, Liotta LA (2002). Clinical proteomics: translating benchside promise into bedside reality. Nat Rev Drug Discov.

[59] Ruzinova MB, Caron T, Rodig SJ (2010). Altered subcellular localization of c-Myc protein identifies aggressive B-cell lymphomas harboring a c-MYC translocation. Am J Surg Pathol.

[60] Green TM, Nielsen O, de Stricker K, Xu-Monette ZY, Young KH, Moller MB (2012). High levels of nuclear MYC protein predict the presence of MYC rearrangement in diffuse large B-cell lymphoma. Am J Surg Pathol.

[61] Johnson NA, Slack GW, Savage KJ, Connors JM, Ben-Neriah S, Rogic S, Scott DW, Tan KL, Steidl C, Sehn LH (2012). Concurrent expression of MYC and BCL2 in diffuse large B-cell lymphoma treated with rituximab plus cyclophosphamide, doxorubicin, vincristine, and prednisone. J Clin Oncol.

[62] Green TM, Young KH, Visco C, Xu-Monette ZY, Orazi A, Go RS, Nielsen O, Gadeberg OV, Mourits-Andersen T, Frederiksen M, Pedersen LM, Moller MB (2012). Immunohistochemical double-hit score is a strong predictor of outcome in patients with diffuse large B-cell lymphoma treated with rituximab plus cyclophosphamide, doxorubicin, vincristine, and prednisone. J Clin Oncol.

[63] Hu S, Xu-Monette ZY, Tzankov A, Green T, Wu L, Balasubramanyam A, Liu WM, Visco C, Li Y, Miranda RN, Montes-Moreno S, Dybkaer K, Chiu A, Orazi A, Zu Y, Bhagat G (2013). MYC/BCL2 protein coexpression contributes to the inferior survival of activated B-cell subtype of diffuse large B-cell lymphoma and demonstrates high-risk gene expression signatures: a report from The International DLBCL Rituximab-CHOP Consortium Program. Blood.

[64] Xu-Monette Z, Dabaja B, Wang X, Tu M, Manyam G, Tzankov A, Xia Y, Li Z, Carlo V, Karen D, Lihui Y, April C, Attilio O, Youli Z, Govind B, Kristy LR (2015). Clinical features, tumor biology and prognosis associated with MYC rearrangement and overexpression in diffuse large B-cell lymphoma patients treated with rituximab-CHOP. Mod Pathol.

[65] Jaffe ES, Pittaluga S (2011). Aggressive B-cell lymphomas: a review of new and old entities in the WHO classification. ASH Education Program Book.

[66] Boerma E, Siebert R, Kluin PM, Baudis M (2009). Translocations involving 8q24 in Burkitt lymphoma and other malignant lymphomas: a historical review of cytogenetics in the light of todays knowledge. Leukemia.

[67] Haralambieva E, Boerma E-J, van Imhoff GW, Rosati S, Schuuring E, Müller-Hermelink HK, Kluin PM, Ott G (2005). Clinical, immunophenotypic, and genetic analysis of adult lymphomas with morphologic features of Burkitt lymphoma. Am J Surg Pathol.

[68] Hummel M, Bentink S, Berger H, Klapper W, Wessendorf S, Barth T, Bernd H, Cogliatti S, Dierlamm J, Feller A (2006). Molecular Mechanisms in Malignant Lymphomas Network Project of the Deutsche Krebshilfe a biologic definition of Burkitt's lymphoma from transcriptional and genomic profiling. N Engl J Med.

[69] Niitsu N, Okamoto M, Miura I, Hirano M (2009). Clinical features and prognosis of de novo diffuse large B-cell lymphoma with t (14; 18) and 8q24/c-MYC translocations. Leukemia.

[70] Bertrand P, Bastard C, Maingonnat C, Jardin F, Maisonneuve C, Courel MN, Ruminy P, Picquenot JM, Tilly H (2007). Mapping of MYC breakpoints in 8q24 rearrangements involving non-immunoglobulin partners in B-cell lymphomas. Leukemia.

[71] Valera A, Balagué O, Colomo L, Martínez A, Delabie J, Taddesse-Heath L, Jaffe ES, Campo E (2010). IG/MYC rearrangements are the main cytogenetic alteration in plasmablastic lymphomas. Am J Surg Pathol.

[72] Onnis A, De Falco G, Antonicelli G, Onorati M, Bellan C, Sherman O, Sayed S, Leoncini L (2010). Alteration of microRNAs regulated by c-Myc in Burkitt lymphoma. PLoS One.

[73] Salaverria I, Martin-Guerrero I, Wagener R, Kreuz M, Kohler CW, Richter J, Pienkowska-Grela B, Adam P, Burkhardt B, Claviez A, Damm-Welk C, Drexler HG, Hummel M, Jaffe ES, Kuppers R, Lefebvre C (2014). A recurrent 11q aberration pattern characterizes a subset of MYC-negative high-grade B-cell lymphomas resembling Burkitt lymphoma. Blood.

[74] Hummel M, Bentink S, Berger H, Klapper W, Wessendorf S, Barth TF, Bernd HW, Cogliatti SB, Dierlamm J, Feller AC, Hansmann ML, Haralambieva E, Harder L, Hasenclever D, Kuhn M, Lenze D (2006). A biologic definition of Burkitt's lymphoma from transcriptional and genomic profiling. N Engl J Med.

[75] Dave SS, Fu K, Wright GW, Lam LT, Kluin P, Boerma EJ, Greiner TC, Weisenburger DD, Rosenwald A, Ott G, Muller-Hermelink HK, Gascoyne RD, Delabie J, Rimsza LM, Braziel RM, Grogan TM (2006). Molecular diagnosis of Burkitt's lymphoma. N Engl J Med.

[76] Dybkaer K, Bogsted M, Falgreen S, Bodker JS, Kjeldsen MK, Schmitz A, Bilgrau AE, Xu-Monette ZY, Li L, Bergkvist KS, Laursen MB, Rodrigo-Domingo M, Marques SC, Rasmussen SB, Nyegaard M, Gaihede M (2015). Diffuse large B-cell lymphoma classification system that associates normal B-cell subset phenotypes with prognosis. J Clin Oncol.

[77] Stasik CJ, Nitta H, Zhang W, Mosher CH, Cook JR, Tubbs RR, Unger JM, Brooks TA, Persky DO, Wilkinson ST (2010). Increased MYC gene copy number correlates with increased mRNA levels in diffuse large B-cell lymphoma. Haematologica.

[78] Pasqualucci L, Trifonov V, Fabbri G, Ma J, Rossi D, Chiarenza A, Wells VA, Grunn A, Messina M, Elliot O (2011). Analysis of the coding genome of diffuse large B-cell lymphoma. Nat Genet.

[79] Valera A, López-Guillermo A, Cardesa-Salzmann T, Climent F, González-Barca E, Mercadal S, Espinosa Í, Novelli S, Briones J, Mate JL (2013). MYC protein expression and genetic alterations have prognostic impact in patients with diffuse large B-cell lymphoma treated with immunochemotherapy. Haematologica.

[80] Horn H, Ziepert M, Becher C, Barth TF, Bernd H-W, Feller AC, Klapper W, Hummel M, Stein H, Hansmann M-L (2013). MYC status in concert with BCL2 and BCL6 expression predicts outcome in diffuse large B-cell lymphoma. Blood.

[81] Tomita N (2011). BCL2 and MYC dual-hit lymphoma/leukemia. J Clin Exp Hematop.

[82] Savage KJ, Johnson NA, Ben-Neriah S, Connors JM, Sehn LH, Farinha P, Horsman DE, Gascoyne RD (2009). MYC gene rearrangements are associated with a poor prognosis in diffuse large B-cell lymphoma patients treated with R-CHOP chemotherapy. Blood.

[83] Barrans S, Crouch S, Smith A, Turner K, Owen R, Patmore R, Roman E, Jack A (2010). Rearrangement of MYC is associated with poor prognosis in patients with diffuse large B-cell lymphoma treated in the era of rituximab. J Clin Oncol.

[84] Bertrand P, Bastard C, Maingonnat C, Jardin F, Maisonneuve C, Courel M, Ruminy P, Picquenot J, Tilly H (2007). Mapping of MYC breakpoints in 8q24 rearrangements involving non-immunoglobulin partners in B-cell lymphomas. Leukemia.

[85] Le Gouill S, Talmant P, Touzeau C, Moreau A, Garand R, Juge-Morineau N, Gaillard F, Gastinne T, Milpied N, Moreau P (2007). The clinical presentation and prognosis of diffuse large B-cell lymphoma with t (14; 18) and 8q24/c-MYC rearrangement. Haematologica.

[86] Snuderl M, Kolman OK, Chen Y-B, Hsu JJ, Ackerman AM, Dal Cin P, Ferry JA, Harris NL, Hasserjian RP, Zukerberg LR (2010). B-cell lymphomas with concurrent IGH-BCL2 and MYC rearrangements are aggressive neoplasms with clinical and pathologic features distinct from Burkitt lymphoma and diffuse large B-cell lymphoma. Am J Surg Pathol.

[87] Li S, Lin P, Fayad LE, Lennon PA, Miranda RN, Yin CC, Lin E, Medeiros LJ (2012). B-cell lymphomas with MYC/8q24 rearrangements and IGH@ BCL2/t (14; 18)(q32; q21): an aggressive disease with heterogeneous histology, germinal center B-cell immunophenotype and poor outcome. Mod Pathol.

[88] Lin P, Dickason TJ, Fayad LE, Lennon PA, Hu P, Garcia M, Routbort MJ, Miranda R, Wang X, Qiao W (2012). Prognostic value of MYC rearrangement in cases of B-cell lymphoma, unclassifiable, with features intermediate between diffuse large B-cell lymphoma and Burkitt lymphoma. Cancer.

[89] Momose S, Weissbach S, Pischimarov J, Nedeva T, Bach E, Rudelius M, Geissinger E, Staiger AM, Ott G, Rosenwald A (2015). The diagnostic gray zone between Burkitt lymphoma and diffuse large B-cell lymphoma is also a gray zone of the mutational spectrum. Leukemia.

[90] Li S, Seegmiller AC, Lin P, Wang XJ, Miranda RN, Bhagavathi S, Medeiros LJ (2015). B-cell lymphomas with concurrent MYC and BCL2 abnormalities other than translocations behave similarly to MYC/BCL2 double-hit lymphomas. Mod Pathol.

[91] Mitelman F, Johansson B, Mertens F (2012). Mitelman database of chromosome aberrations and gene fusions in cancer.

[92] Aukema SM, Siebert R, Schuuring E, van Imhoff GW, Kluin-Nelemans HC, Boerma E-J, Kluin PM (2011). Double-hit B-cell lymphomas. Blood.

[93] Pillai RK, Sathanoori M, Van Oss SB, Swerdlow SH (2013). Double-hit B-cell lymphomas with BCL6 and MYC translocations are aggressive, frequently extranodal lymphomas distinct from BCL2 double-hit B-cell lymphomas. Am J Surg Pathol.

[94] Wang W, Hu S, Lu X, Young KH, Medeiros LJ (2015). Triple-hit B-cell Lymphoma With MYC, BCL2, and BCL6 Translocations/Rearrangements: Clinicopathologic Features of 11 Cases. Am J Surg Pathol.

[95] Kuehl WM, Bergsagel PL (2012). Molecular pathogenesis of multiple myeloma and its premalignant precursor. J Clin Invest.

[96] Avet-Loiseau H, Gerson F, Magrangeas F, Minvielle S, Harousseau J-L, Bataille R (2001). Rearrangements of the c-myc oncogene are present in 15% of primary human multiple myeloma tumors. Blood.

[97] Gabrea A, Martelli ML, Qi Y, Roschke A, Barlogie B, Shaughnessy JD, Sawyer JR, Kuehl WM (2008). Secondary genomic rearrangements involving immunoglobulin or MYC loci show similar prevalences in hyperdiploid and nonhyperdiploid myeloma tumors. Genes Chromosomes Cancer.

[98] Taddesse-Heath L, Meloni-Ehrig A, Scheerle J, Kelly JC, Jaffe ES (2010). Plasmablastic lymphoma with MYC translocation: evidence for a common pathway in the generation of plasmablastic features. Mod Pathol.

[99] Ouansafi I, He B, Fraser C, Nie K, Mathew S, Bhanji R, Hoda R, Arabadjief M, Knowles D, Cerutti A (2010). Transformation of follicular lymphoma to plasmablastic lymphoma with c-myc gene rearrangement. Am J Clin Pathol.

[100] Martinez D, Valera A, Perez NS, Villegas LFS, Gonzalez-Farre B, Sole C, Gine E, Lopez-Guillermo A, Roue G, Martinez S (2013). Plasmablastic transformation of low-grade B-cell lymphomas: report on 6 cases. Am J Surg Pathol.

[101] Hart LS, Cunningham JT, Datta T, Dey S, Tameire F, Lehman SL, Qiu B, Zhang H, Cerniglia G, Bi M (2012). ER stress-mediated autophagy promotes Myc-dependent transformation and tumor growth. J Clin Invest.

[102] Cortelazzo S, Ponzoni M, Ferreri AJ, Hoelzer D (2011). Lymphoblastic lymphoma. Crit Rev Oncol/Hematol.

[103] Nathwani BN, Diamond LW, Winberg CD, Kim H, Bearman RM, Glick JH, Jones SE, Gams RA, Nissen NI, Rappaport H (1981). Lymphoblastic lymphoma: a clinicopathologic study of 95 patients. Cancer.

[104] Groves FD, Linet MS, Travis LB, Devesa SS (2000). Cancer surveillance series: non-Hodgkin's lymphoma incidence by histologic subtype in the United States from 1978 through 1995. J Natl Cancer Inst.

[105] Soslow RA, Baergen RN, Warnke RA (1999). B-lineage lymphoblastic lymphoma is a clinicopathologic entity distinct from other histologically similar aggressive lymphomas with blastic morphology. Cancer.

[106] Weng AP, Millholland JM, Yashiro-Ohtani Y, Arcangeli ML, Lau A, Wai C, del Bianco C, Rodriguez CG, Sai H, Tobias J (2006). c-Myc is an important direct target of Notch1 in T-cell acute lymphoblastic leukemia/lymphoma. Genes Dev.

[107] Weng AP, Ferrando AA, Lee W, Morris JP, Silverman LB, Sanchez-Irizarry C, Blacklow SC, Look AT, Aster JC (2004). Activating mutations of NOTCH1 in human T cell acute lymphoblastic leukemia. Science.

[108] Ferrando AA, Neuberg DS, Staunton J, Loh ML, Huard C, Raimondi SC, Behm FG, Pui C-H, Downing JR, Gilliland DG (2002). Gene expression signatures define novel oncogenic pathways in T cell acute lymphoblastic leukemia. Cancer cell.

[109] Pear WS, Aster JC, Scott ML, Hasserjian RP, Soffer B, Sklar J, Baltimore D (1996). Exclusive development of T cell neoplasms in mice transplanted with bone marrow expressing activated Notch alleles. J Exp Med.

[110] Young KH, Xie Q, Zhou G, Eickhoff JC, Sanger WG, Aoun P, Chan WC (2008). Transformation of follicular lymphoma to precursor B-cell lymphoblastic lymphoma with c-myc gene rearrangement as a critical event. Am J Clin Pathol.

[111] Strasser A, Harris AW, Bath ML, Cory S (1990). Novel primitive lymphoid tumours induced in transgenic mice by cooperation between myc and bcl-2. Nature.

[112] Feng H, Stachura DL, White RM, Gutierrez A, Zhang L, Sanda T, Jette CA, Testa JR, Neuberg DS, Langenau DM (2010). T-lymphoblastic lymphoma cells express high levels of BCL2, S1P1, and ICAM1, leading to a blockade of tumor cell intravasation. Cancer cell.

[113] Davies AJ, Rosenwald A, Wright G, Lee A, Last KW, Weisenburger DD, Chan WC, Delabie J, Braziel RM, Campo E (2007). Transformation of follicular lymphoma to diffuse large B-cell lymphoma proceeds by distinct oncogenic mechanisms. Br J Haematol.

[114] Pasqualucci L, Khiabanian H, Fangazio M, Vasishtha M, Messina M, Holmes AB, Ouillette P, Trifonov V, Rossi D, Tabbo F, Ponzoni M, Chadburn A, Murty VV, Bhagat G, Gaidano G, Inghirami G (2014). Genetics of follicular lymphoma transformation. Cell Rep.

[115] Lossos IS, Alizadeh AA, Diehn M, Warnke R, Thorstenson Y, Oefner PJ, Brown PO, Botstein D, Levy R (2002). Transformation of follicular lymphoma to diffuse large-cell lymphoma: alternative patterns with increased or decreased expression of c-myc and its regulated genes. Proc Natl Acad Sci U S A.

[116] Glas AM, Kersten MJ, Delahaye LJ, Witteveen AT, Kibbelaar RE, Velds A, Wessels LF, Joosten P, Kerkhoven RM, Bernards R, van Krieken JH, Kluin PM, van't Veer LJ, de Jong D (2005). Gene expression profiling in follicular lymphoma to assess clinical aggressiveness and to guide the choice of treatment. Blood.

[117] Gatenby RA, Gillies RJ (2004). Why do cancers have high aerobic glycolysis?. Nat Rev Cancer.

[118] Amin HM, McDonnell TJ, Medeiros LJ, Rassidakis GZ, Leventaki V, O'Connor SL, Keating MJ, Lai R (2003). Characterization of 4 mantle cell lymphoma cell lines. Arch Pathol Lab Med.

[119] Lai R, Medeiros LJ (2000). Pathologic diagnosis of mantle cell lymphoma. Clin Lymphoma.

[120] Hao S, Sanger W, Onciu M, Lai R, Schlette EJ, Medeiros LJ (2002). Mantle cell lymphoma with 8q24 chromosomal abnormalities: a report of 5 cases with blastoid features. Mod Pathol.

[121] Sanchez R, Zhou M-M (2009). The role of human bromodomains in chromatin biology and gene transcription. Curr Opin Drug Discov Devel.

[122] Wilson WH (2013). Treatment strategies for aggressive lymphomas: what works?. ASH Education Program Book.

[123] Furqan M, Akinleye A, Mukhi N, Mittal V, Chen Y, Liu D (2013). STAT inhibitors for cancer therapy. J Hematol Oncol.

[124] Savino M, Annibali D, Carucci N, Favuzzi E, Cole MD, Evan GI, Soucek L, Nasi S (2011). The action mechanism of the Myc inhibitor termed Omomyc may give clues on how to target Myc for cancer therapy. PLoS One.

[125] Ott CJ, Kopp N, Bird L, Paranal RM, Qi J, Bowman T, Rodig SJ, Kung AL, Bradner JE, Weinstock DM (2012). BET bromodomain inhibition targets both c-Myc and IL7R in high-risk acute lymphoblastic leukemia. Blood.

[126] Chaidos A, Caputo V, Karadimitris A (2015). Inhibition of bromodomain and extra-terminal proteins (BET) as a potential therapeutic approach in haematological malignancies: emerging preclinical and clinical evidence. Ther Adv Hematol.

[127] Le A, Cooper CR, Gouw AM, Dinavahi R, Maitra A, Deck LM, Royer RE, Vander Jagt DL, Semenza GL, Dang CV (2010). Inhibition of lactate dehydrogenase A induces oxidative stress and inhibits tumor progression. Proc Natl Acad Sci USA.

[128] Wahl ML, Owen JA, Burd R, Herlands RA, Nogami SS, Rodeck U, Berd D, Leeper DB, Owen CS (2002). Regulation of Intracellular pH in Human Melanoma: Potential Therapeutic Implications. Mol Cancer Ther.

[129] Qing G, Li B, Vu A, Skuli N, Walton ZE, Liu X, Mayes PA, Wise DR, Thompson CB, Maris JM (2012). ATF4 regulates MYC-mediated neuroblastoma cell death upon glutamine deprivation. Cancer Cell.

[130] Li B, Simon MC (2013). Molecular Pathways: Targeting MYC-induced metabolic reprogramming and oncogenic stress in cancer. Clin Cancer Res.

[131] Rahl PB, Lin CY, Seila AC, Flynn RA, McCuine S, Burge CB, Sharp PA, Young RA (2010). c-Myc regulates transcriptional pause release. Cell.

[132] Goga A, Yang D, Tward AD, Morgan DO, Bishop JM (2007). Inhibition of CDK1 as a potential therapy for tumors over-expressing MYC. Nat Med.

[133] Yu B, Lane ME, Wadler S (2002). SU9516, a cyclin-dependent kinase 2 inhibitor, promotes accumulation of high molecular weight E2F complexes in human colon carcinoma cells. Biochem Pharmacol.

[134] Montagnoli A, Valsasina B, Croci V, Menichincheri M, Rainoldi S, Marchesi V, Tibolla M, Tenca P, Brotherton D, Albanese C, Patton V, Alzani R, Ciavolella A, Sola F, Molinari A, Volpi D (2008). A Cdc7 kinase inhibitor restricts initiation of DNA replication and has antitumor activity. Nat Chem Biol.

[135] Chen R, Wierda WG, Chubb S, Hawtin RE, Fox JA, Keating MJ, Gandhi V, Plunkett W (2009). Mechanism of action of SNS-032, a novel cyclin-dependent kinase inhibitor, in chronic lymphocytic leukemia. Blood.

[136] Filippakopoulos P, Qi J, Picaud S, Shen Y, Smith WB, Fedorov O, Morse EM, Keates T, Hickman TT, Felletar I (2010). Selective inhibition of BET bromodomains. Nature.

[137] Zippo A, De Robertis A, Serafini R, Oliviero S (2007). PIM1-dependent phosphorylation of histone H3 at serine 10 is required for MYC-dependent transcriptional activation and oncogenic transformation. Nat Cell Biol.

[138] Zippo A, Serafini R, Rocchigiani M, Pennacchini S, Krepelova A, Oliviero S (2009). Histone crosstalk between H3S10ph and H4K16ac generates a histone code that mediates transcription elongation. Cell.

[139] Daigle SR, Olhava EJ, Therkelsen CA, Majer CR, Sneeringer CJ, Song J, Johnston LD, Scott MP, Smith JJ, Xiao Y (2011). Selective killing of mixed lineage leukemia cells by a potent small-molecule DOT1L inhibitor. Cancer Cell.

[140] McMahon SB, Van Buskirk HA, Dugan KA, Copeland TD, Cole MD (1998). The novel ATM-related protein TRRAP is an essential cofactor for the c-Myc and E2F oncoproteins. Cell.

[141] Xu Y, Voelter-Mahlknecht S, Mahlknecht U (2005). The histone deacetylase inhibitor suberoylanilide hydroxamic acid down-regulates expression levels of Bcr-abl, c-Myc and HDAC3 in chronic myeloid leukemia cell lines. Int J Mol Med.

[142] Titov DV, Gilman B, He QL, Bhat S, Low WK, Dang Y, Smeaton M, Demain AL, Miller PS, Kugel JF, Goodrich JA, Liu JO (2011). XPB, a subunit of TFIIH, is a target of the natural product triptolide. Nat Chem Biol.

[143] Felsher DW, Zetterberg A, Zhu J, Tlsty T, Bishop JM (2000). Overexpression of MYC causes p53-dependent G2 arrest of normal fibroblasts. Proc Natl Acad Sci USA.

[144] Campas C, López JM, Santidrián AF, Barragán M, Bellosillo B, Colomer D, Gil J (2003). Acadesine activates AMPK and induces apoptosis in B-cell chronic lymphocytic leukemia cells but not in T lymphocytes. Blood.

[145] Ikezoe T, Nishioka C, Bandobashi K, Yang Y, Kuwayama Y, Adachi Y, Takeuchi T, Koeffler HP, Taguchi H (2007). Longitudinal inhibition of PI3K/Akt/mTOR signaling by LY294002 and rapamycin induces growth arrest of adult T-cell leukemia cells. Leuk Res.

[146] Distler A, Deloch L, Huang J, Dees C, Lin NY, Palumbo-Zerr K, Beyer C, Weidemann A, Distler O, Schett G, Distler JH (2013). Inactivation of tankyrases reduces experimental fibrosis by inhibiting canonical Wnt signalling. Ann Rheum Dis.

[147] Moellering RE, Cornejo M, Davis TN, Del Bianco C, Aster JC, Blacklow SC, Kung AL, Gilliland DG, Verdine GL, Bradner JE (2009). Direct inhibition of the NOTCH transcription factor complex. Nature.

[148] Yang D, Liu H, Goga A, Kim S, Yuneva M, Bishop JM (2010). Therapeutic potential of a synthetic lethal interaction between the MYC proto-oncogene and inhibition of aurora-B kinase. Proc Natl Acad Sci U S A.

[149] Zhang XY, Pfeiffer HK, Mellert HS, Stanek TJ, Sussman RT, Kumari A, Yu D, Rigoutsos I, Thomas-Tikhonenko A, Seidel HE, Chodosh LA, Packham G, Baserga R, McMahon SB (2011). Inhibition of the single downstream target BAG1 activates the latent apoptotic potential of MYC. Mol Cell Biol.

[150] Mason KD, Vandenberg CJ, Scott CL, Wei AH, Cory S, Huang DC, Roberts AW (2008). *In vivo* efficacy of the Bcl-2 antagonist ABT-737 against aggressive Myc-driven lymphomas. Proc Natl Acad Sci USA.

[151] Goloudina AR, Demidov ON, Garrido C (2012). Inhibition of HSP70: a challenging anti-cancer strategy. Cancer Lett.

[152] Nakashima T, Ishii T, Tagaya H, Seike T, Nakagawa H, Kanda Y, Akinaga S, Soga S, Shiotsu Y (2010). New molecular and biological mechanism of antitumor activities of KW-2478, a novel nonansamycin heat shock protein 90 inhibitor, in multiple myeloma cells. Clin Cancer Res.

[153] Murga M, Campaner S, Lopez-Contreras AJ, Toledo LI, Soria R, Montana MF, D'Artista L, Schleker T, Guerra C, Garcia E, Barbacid M, Hidalgo M, Amati B, Fernandez-Capetillo O (2011). Exploiting oncogene-induced replicative stress for the selective killing of Myc-driven tumors. Nat Struct Mol Biol.

[154] Baumann P, Mandl-Weber S, Volkl A, Adam C, Bumeder I, Oduncu F, Schmidmaier R (2009). Dihydroorotate dehydrogenase inhibitor A771726 (leflunomide) induces apoptosis and diminishes proliferation of multiple myeloma cells. Mol Cancer Ther.

[155] Page BD, Khoury H, Laister RC, Fletcher S, Vellozo M, Manzoli A, Yue P, Turkson J, Minden MD, Gunning PT (2012). Small molecule STAT5-SH2 domain inhibitors exhibit potent antileukemia activity. J Med Chem.

[156] Cuccuini W, Briere J, Mounier N, Voelker H-U, Rosenwald A, Sundstrom C, Cogliatti S, Hirchaud E, Ysebaert L, Bron D (2012). MYC+ diffuse large B-cell lymphoma is not salvaged by classical R-ICE or R-DHAP followed by BEAM plus autologous stem cell transplantation. Blood.

[157] Petrich AM, Gandhi M, Jovanovic B, Castillo JJ, Rajguru S, Yang DT, Shah KA, Whyman JD, Lansigan F, Hernandez-Ilizaliturri FJ, Lee LX, Barta SK, Melinamani S, Karmali R, Adeimy C, Smith S (2014). Impact of induction regimen and stem cell transplantation on outcomes in double-hit lymphoma: a multicenter retrospective analysis. Blood.

[158] Howlett C, Snedecor SJ, Landsburg DJ, Svoboda J, Chong EA, Schuster SJ, Nasta SD, Feldman T, Rago A, Walsh KM, Weber S, Goy A, Mato A (2015). Front-line, dose-escalated immunochemotherapy is associated with a significant progression-free survival advantage in patients with double-hit lymphomas: a systematic review and meta-analysis. Br J Haematol.

[159] Dunleavy K, Pittaluga S, Shovlin M, Pack S, Steinberg SM, Lai CE, Grant C, Hsi ED, Staudt LM, Jaffe ES (2013). Concurrent Expression Of MYC/BCL2 Protein In Newly Diagnosed DLBCL Is Not Associated With An Inferior Survival Following EPOCH-R Therapy. Blood.

[160] Oki Y, Noorani M, Lin P, Davis RE, Neelapu SS, Ma L, Ahmed M, Rodriguez MA, Hagemeister FB, Fowler N (2014). Double hit lymphoma: the MD Anderson Cancer Center clinical experience. Br J Haematol.

[161] Testoni M, Zucca E, Young KH, Bertoni F (2015). Genetic lesions in diffuse large B-cell lymphomas. Ann Oncol.

[162] Young KH, Medeiros LJ, Chan WC, Orazi A, Weiss LM, Foucar K, Knowles DM (2014). Diffuse large B-cell lymphoma. Neoplastic Hematopathology.

